# A polytherapy approach demonstrates therapeutic efficacy for the treatment of *SOD1* associated amyotrophic lateral sclerosis

**DOI:** 10.1016/j.ebiom.2025.105692

**Published:** 2025-04-12

**Authors:** Jeremy S. Lum, Mikayla L. Brown, Natalie E. Farrawell, Rachael Bartlett, Christen G. Chisholm, Jody Gorman, Anthony Dosseto, Florian Dux, Lachlan E. McInnes, Heath Ecroyd, Luke McAlary, Peter J. Crouch, Paul S. Donnelly, Justin J. Yerbury

**Affiliations:** a Molecular Horizons and School of Science, University of Wollongong, Wollongong, NSW 2522, Australia; b School of Medical, Indigenous and Health Sciences, University of Wollongong, Wollongong, NSW 2522, Australia; c Wollongong Isotope Geochronology Laboratory, School of Earth, Atmospheric and Life Sciences, University of Wollongong, Wollongong, NSW, Australia; d School of Chemistry and Bio21 Molecular Science and Biotechnology Institute, University of Melbourne, Parkville, VIC, Australia; e Department of Anatomy and Physiology, Centre for Muscle Research, University of Melbourne, Parkville, VIC 3010, Australia

**Keywords:** SOD1, Amyotrophic lateral sclerosis, Motor neuron disease, Polytherapy, CuATSM, Ebselen, Telbivudine

## Abstract

**Background:**

*SOD1* mutations are a significant contributor of familial amyotrophic lateral sclerosis (ALS) cases. *SOD1* mutations increase the propensity for the protein to misfold and aggregate into insoluble proteinaceous deposits within motor neurons and neighbouring cells. The small molecule, CuATSM, has repeatedly shown in mouse models to be a promising therapeutic treatment for *SOD1*-associated ALS and is currently in Phase II/III clinical trials for the treatment of ALS. We have previously shown CuATSM stabilises various ALS-associated variants of the SOD1 protein, reducing misfolding and toxicity. Two additional FDA-approved small molecules, ebselen and telbivudine, have also been identified to reduce mutant SOD1 toxicity, providing additional potential therapeutic candidates that could be used in combination with CuATSM. Here, we aimed to investigate if CuATSM, ebselen and telbivudine (CET) polytherapy could improve on the therapeutic efficacy of CuATSM monotherapy for the treatment of *SOD1*-associated ALS.

**Methods:**

We utilised a 3D checkerboard approach to investigate whether a matrix of different concentrations CuATSM, ebselen and telbivudine could provide therapeutic improvements on cell survival, SOD1 folding and aggregation in SOD1^G93A^-transfected NSC-34 cells, compared to CuATSM alone. To progress the preclinical development of CET polytherapy, we evaluated the bioavailability and safety of *in vivo* polytherapy administration. Furthermore, we assessed and compared the effects of CET- and CuATSM-treatment on disease onset, motor function, survival and neuropathological features in SOD1^G93A^ mice.

**Findings:**

CET polytherapy reduced inclusion formation and increased cell survival of NSC-34 cells overexpressing SOD1^G93A^ compared to higher concentrations of CuATSM monotherapy. In addition, CET administration was bioavailable and tolerable in mice. CET treatment in SOD1^G93A^ mice delayed disease onset, reduced motor impairments, and increased survival compared to vehicle- and CuATSM-treated mice. In line with these findings, biochemical analysis of lumbar spinal cords showed CET administration improved SOD1 folding, decreased misfolded SOD1 accumulation, and reduced motor neuron loss.

**Interpretation:**

These findings support CET polytherapy as an advantageous alternative compared to CuATSM monotherapy and highlight the potential of utilising small molecules targeting SOD1 as a polytherapy avenue for the treatment of *SOD1*-associated ALS.

**Funding:**

This work was supported by a FightMND Drug Development Grant, an Australian National Health and Medical Research Council (NHMRC) Investigator Grant (No. 1194872) and a Motor Neuron Disease Research Institute of Australia Bill Gole Postdoctoral Fellowship.

**Research in context**

**Evidence before this study**

Superoxide dismutase 1 (SOD1) mutations cause amyotrophic lateral sclerosis (ALS), a fatal neurodegenerative disease. SOD1 mutations destabilise the protein, causing the accumulation of toxic misfolded and aggregated species. The compound, CuATSM, is currently in Phase II/III clinical trials after repeated preclinical reports that CuATSM treatment slows disease progression and improves survival in several mouse models of *SOD1*-associated ALS. CuATSM has shown to work in-part by aiding correct folding of mutant SOD1 and reduce aggregate formation. Two additional FDA-approved small molecules, ebselen and telbivudine, have each shown to aid SOD1 folding and reduce SOD1 toxicity. This provides the opportunity to investigate if ebselen and telbivudine could provide therapeutic advantages when used as a polytherapy with CuATSM for the treatment of SOD1-associated ALS.

**Added value of this study**

In this work, we provide *in vitro* evidence that the therapeutic efficacy of CuATSM is improved when used as a polytherapy with ebselen and telbivudine in a model of SOD1-associated ALS. CuATSM, ebselen and telbivudine (CET) polytherapy improves SOD1 folding, reduces inclusion formation and increases cell survival compared to CuATSM monotherapy. Oral CET polytherapy (CuATSM: 50 mg/kg/day; ebselen: 100 mg/kg/day; telbivudine: 150 mg/kg/day) administration in mice is bioavailable and shows acceptable safety characteristics. Furthermore, CET administration demonstrates superior therapeutic efficacy and neuroprotective properties than CuATSM treatment monotherapy in a mouse model of SOD1-associated ALS.

**Implications of all the available evidence**

Our findings demonstrate that the therapeutic benefits of CuATSM can be improved when used as a polytherapy in conjunction with ebselen and telbivudine for SOD1-associated ALS. This work highlights the potential of utilising multiple small molecules that aid mutant SOD1 folding and/or reduce SOD1 toxicity as a therapeutic avenue for SOD1-associated ALS.

## Introduction

Amyotrophic lateral sclerosis (ALS) is a fatal motor neuron disease resulting in the death of motor neurons and other neuronal populations. The underlying cause(s) of most cases of ALS are undefined and classified as sporadic (sALS). However, approximately 10% of cases are inherited (familial ALS; fALS).^1^ The earliest studied fALS cases were from families possessing *superoxide dismutase-1* (*SOD1*) mutations. There have now been >230 ALS-associated mutations identified in the *SOD1* gene. In different populations 7–23% of diagnosed fALS cases and 1–5% of all ALS cases carries a SOD1 mutation.^2^ Under normal physiological condition, the SOD1 protein acts as a cytosolic free radical scavenger. ALS-associated *SOD1* mutations increase the propensity for the SOD1 protein to misfold and form neurotoxic protein aggregates that underlies a potential gain-of-function pathology.^3^

Nascent SOD1 undergoes extensive post-translational modifications in the process of folding into its native dimeric form. Newly synthesised SOD1 forms a partially-folded state that facilitates spontaneous binding of Zn^2+^.^4^ Subsequently, the copper chaperone for SOD1 (CCS), delivers Cu^2+^ to SOD1, leading to the intramolecular disulfide bond becoming oxidised, and mature SOD1 monomer homodimerisation.^5^ ALS-associated mutations within *SOD1* alters the physical properties of the SOD1 protein to various degrees, causing mutant SOD1 to diverge towards off-folding pathways at multiple stages, resulting in varying levels of toxicity. This has led to the hypothesis that small molecules that can simultaneously aid mutant SOD1 folding, increase folding stability, and prevent the divergence towards off-folding pathways could potentially be employed as a treatment strategy for *SOD1*-associated ALS.^6^

Diacetyl*bis*(*N*(4)-methylthiosemicarbazonato)copper(II) (CuATSM) is a stable, low molecular weight, lipophilic compound that has shown to cross the blood brain barrier.^7^^,^^8^ CuATSM is currently in Phase II/III clinical trials in Australia (NCT04082832) after repeated reports that CuATSM treatment slows disease progression and improves survival in several mouse models of *SOD1*-associated ALS.^8^, ^9^, ^10^, ^11^, ^12^, ^13^ CuATSM has been shown to act via several mechanisms, including reducing peroxynitrate levels,^8^ restoring mitochondrial function,^14^^,^^15^ and having anti-ferroptotic activity.^16^^,^^17^ Furthermore, CuATSM can increase CCS-mediated Cu^2+^ delivery to SOD1.^18^ We have previously found that *in vitro* CuATSM treatment reduces SOD1 aggregation and increases cell survival for *SOD1* mutants that retain enzymatic activity similar to the wild type protein (herein referred to wild type-like (WTL) *SOD1* mutants). However, we showed that CuATSM provided little-to-no benefit for metal binding region (MBR) *SOD1* mutations, in which Cu^2+^ binding is disrupted.^19^^,^^20^ Our previous results suggest approaches that promote Cu^2+^ binding, subsequently increasing SOD1 maturation, may be an attractive target for WTL-*SOD1* mutations, whilst MBR*-SOD1* mutations may require alternative strategies to promote maturation.

With the aim of identifying additional small molecules capable of aiding SOD1 folding, we and others demonstrated that the seleno-organic compound, ebselen, promotes SOD1 intramolecular disulfide bond formation and SOD1 homodimer affinity.^19^^,^^21^^,^^22^ ALS-associated *SOD1* mutations of Cys57 and Cys146, residues responsible for disulfide bonding, are rare, thereby making ebselen a suitable molecule to promote SOD1 folding for a majority of *SOD1* mutations. Moreover, we have demonstrated ebselen in the absence or presence of CuATSM, increases SOD1 maturation, reduces inclusion formation, and increases survival of cultured neuronal-like NSC-34 cells over-expressing either WTL- and MBR-SOD1 mutant isoforms.^19^ This work further supports the concept that aiding SOD1 maturation is a potential therapeutic approach for *SOD1*-associated ALS and that polytherapy may be more effective than a monotherapy, particularly in regards to CuATSM, which has reached clinical trials.

Whilst reducing the levels of SOD1 protein that diverge towards off-folding pathways is potentially useful, there is also a need to target the population of mutant SOD1 that may evade intervention and aggregate. Recently, pyrimidine-based compounds, have been shown to reduce SOD1-induced toxicity in cultured cells and zebrafish.^23^^,^^24^ Interestingly, the pyrimidine-based antiviral compound, telbivudine, which has been used to treat chronic hepatitis B, was found to attenuate axonopathy in a zebrafish model of *SOD1*-associated ALS.^23^ It has been postulated that telbivudine interacts with Trp32 within SOD1, which has been shown to be play a critical role in facilitating the aggregation and prion-like propagation of mutant SOD1.^24^, ^25^, ^26^

Polytherapies have become one of the most significant advances in the treatment of cancer and have been demonstrated to have many advantages over traditional monotherapy approaches. This includes the ability to target multiple aspects of pathology, provide synergistic or additive actions and the potential to use lower therapeutic doses of drugs to reduce adverse events. Whilst the therapeutic benefits of CuATSM, ebselen and telbivudine monotherapies, as well as CuATSM and ebselen polytherapy, have been demonstrated to rescue SOD1 toxicity in cell and mouse models of SOD1-associated ALS, the potential of a polytherapy incorporating all three compounds together has yet to be investigated. Here, we demonstrate using an *in vitro* cell model of *SOD1*-associated ALS that CuATSM, ebselen and telbivudine (CET) polytherapy improves cell survival, decreases SOD1 inclusion formation and improves SOD1 folding compared to CuATSM alone. Similarly, in the well-characterised SOD1^G93A^ mouse model of SOD1 ALS, CET polytherapy delayed disease onset and increased survival compared to CuATSM administration alone. This work highlights the potential of targeting multiple aspects of the SOD1 folding pathway as a therapeutic treatment avenue for *SOD1*-associated ALS.

## Methods

### Plasmids

pEGFP-N1 vector containing human SOD1^G93A^ was generated as previously described.^27^ The SOD1^G93A^-tdTomato construct was generated by replacing the enhanced green fluorescent protein (GFP) sequence in pEGFP-N1-SOD1^G93A^ with the tomato red fluorescent protein.

### Cell culture and transfection

Neuroblastoma × spinal cord hybrid NSC-34 cells (RRID:CVCL_D356)^28^ were maintained in Dulbecco’s Modified Eagle’s Medium/Ham’s Nutrient Mixture F12 (DMEM/F12) supplemented with 10% (v/v) foetal bovine serum (FBS, Gibco, Australia, #10091148). Cells were maintained at 37 °C in a humidified incubator with 5% atmospheric CO_2_. Cells were plated into 6-well plates 24 h prior to transfection. Cells were transfected with 2.5 μg of DNA (per well) using Lipofectamine 3000 (Invitrogen, #L3000075), according to the manufacturer’s instructions. Approximately 5 h post transfection, cells were replated at 30% confluency into either 96-well plates to monitor cell survival, or 12-well plates to generate cell lysates. Treatment of transfected cells with drugs commenced 24 h post transfection.

### 3D checkerboard assay

A 3D checkboard assay was performed to establish the optimal ratio and concentration of CET treatment to use on SOD1^G93A^-transfected NSC-34 cells. Firstly, stock solutions of CuATSM (300 μM), ebselen (12 mM) and telbivudine (300 mM) were prepared in dimethyl sulfoxide (DMSO). Stocks solutions were then used to create intermediate serial dilutions of each drug (0–3 μM CuATSM, 0–120 μM ebselen and 0–3000 μM telbivudine) in DMEM/F12 supplemented with 10% FBS with a final DMSO concentration of 1% (v/v). These dilutions were pipetted down (CuATSM), across (ebselen), and over (telbivudine), multiple 96-well plates in a schematic similar to that outlined in,^29^ so that when they were added at a 1:1 (v/v) ratio with cells, the final concentrations of each drug were 0–0.5 μM CuATSM, 0–20 μM ebselen and 0–500 μM telbivudine in 0.5% (v/v) DMSO.

### Monitoring cell survival

The survival of NSC-34 cells expressing SOD1^G93A^-EGFP and treated with either CuATSM or CET (or DMSO vehicle control) treatment was monitored over 48 h in an Incucyte S3 automated fluorescent microscope following methods previously described.^30^ Briefly, images of cells within each well of the plates were acquired every 3 h using a 10 × objective in both phase and green channels with the green channel acquisition time set at 300 ms. Cell images were analysed using a processing definition trained to select GFP-positive cells (Top-Hat segmentation, 2 Green Calibrated Unit (GCU) threshold adjustment, edge splitting, hole fill clean-up 60 μm^2^ and minimum area filter of 200 μm^2^). To compare the survival of cells across treatments, the number of GFP-positive cells were counted in each image and normalised to the count at time zero, before normalising to the vehicle-treated control values.

### Image analysis to quantify SOD1 inclusion formation

Analysis of the images generated from the automated live-cell imaging of SOD1^G93A^-expressing cells on the IncuCyteS3 was performed as described,^31^ with the following exceptions. Illumination correction of images was performed using the Smooth and ImageMath modules in CellProfiler. Firstly, a gaussian filter with a typical artefact diameter of 64 pixels was applied to all GFP images to create a filtered image. This filtered image was then subtracted from the original GFP image using the ImageMath module. Following this illumination correction step, images were processed in CellProfiler to segment cells with a typical diameter between 10 and 30 pixel units and measure granularity, texture, size/shape, intensity distribution, and intensity. The thumbnails generated by this pipeline were subsequently processed in CellProfiler Analyst and machine learning with a random forest classifier was trained to differentiate between cells with and without inclusions using at least 300 cells per bin.^31^ Following successful training (accuracy above 95%) the entire dataset of cells was classified.

### Immunoblotting for *in vitro* SOD1 di-sulfide formation

Immunoblotting of cell lysates was performed as described.^19^ In summary, NSC-34 cells transfected with SOD1^G93A^-EGFP cells were lysed 8 h post drug treatment in ice-cold 1 × Tris-buffered saline (pH 7.4) with 1% Trition X-100 (v/v) and 1 mg/ml N-ethylmaleimide supplemented with 1 × Halt protease inhibitor (Thermo Fisher Scientific, #78429) and mixed 1:3 with either 4 × non-reducing SDS-PAGE sample buffer or 4 × reducing SDS-PAGE sample buffer (containing 2.5% 2-mercaptoethanol (v/v)) for SDS-PAGE gel electrophoresis. Following electrophoresis, protein was transferred onto methanol-activated Amersham Hybond 0.2 μm polyvinylidene difluoride membranes (GE Healthcare). Membranes were blocked with in Tris-buffered saline containing 0.02% Tween 20 (w/v) (TBST) and 5% skim milk powder for 1 h at room temperature and subsequently probed for SOD1-GFP using polyclonal rabbit anti-GFP primary antibody (Abcam, ab290, RRID:AB_303395, 1:10,000) overnight in blocking solution. Following primary antibody incubation, membranes were washed in Tris-buffered saline with 0.1% Tween-20 (v/v) (TBST) and incubated in goat anti-rabbit horseradish peroxidase–conjugated secondary antibody (DAKO, P0448, RRID:AB_2617138, 1:5000), before detection with SuperSignal West Pico Plus substrate (Thermo Fisher Scientific, #34580). Imaging was performed on a ChemiDoc MP Imaging System (Bio-Rad) and analysis and quantification performed using ImageJ (version 1.53c, National Institutes of Health).^32^

### In-gel zymography to measure SOD1 activity *in vitro*

NSC-34 cells transfected with SOD1^G93A^-tdTomato were harvested 48 h post drug treatment in 100 mM Tris-base (pH 7.5) with 0.1% Trition X-100 (v/v) and protease inhibitor cocktail, before native-PAGE was performed as described previously.^19^ Following native-PAGE, the TdTomato signal in the gel was detected using a ChemiDoc MP Imaging System (Bio-Rad), and gels were subjected to zymography as described previously.^33^ Quantification of tdTomato fluorescence and enzymatic activity was performed using ImageJ (version 1.53c, National Institutes of Health).

### Ethics

All animal experiments were approved by the University of Wollongong Animal Ethics Committee (approval number: AEPR21/15) and complied with the National Health and Medical Research Institute, Australian Code of Practice for the Care and Use of Animals and Scientific Purposes. This study is reported in accordance with the Animal Research: Reporting of *in vivo* Experiments (ARRIVE) guidelines.

### Animals

The number of animal numbers allocated to each treatment group was based on recommendations from recommendations of the ALS Therapy Development Institute.^34^ Male and female heterozygous human SOD1^G93A^ mice (B6-Tg(SOD1-G93A)1GUr/j); The Jackson Laboratory Stock Number: 004435] were maintained on a C57BL/6J background and bred at the Australian Bioresources Animal Facility (Moss Vale, Australia). Pups were weaned and genotyped at approximately postnatal day 21–28. Genotype confirmed SOD1^G93A^ mice were transported to the University of Wollongong. Mice were housed with littermates where possible, with 2–4 females or 2–4 males per cage. Mice were housed in IVC cages (Greenline GM500, Techniplast, Australia) under a 12:12 h light–dark cycle (illuminated from 0700 to 1900 h). IVC cages included a layering of iso-PADS™ bedding (Envigo), tissue, Bed r’Nest™ (Techniplast, Australia), a plastic house and a PVC tunnel. Food and water were available ad libitum. Once mice reached 100 days old, water-soaked food pellets were placed on the cage floor and longer sippers placed on water bottles.

### Drug preparation

CuATSM was synthesised and prepared as previously described.^35^ Ebselen and telbivudine were purchased from SYNthesis med chem (Melbourne, Australia). CuATSM or CET combination therapy; CuATSM, ebselen and telbivudine, was suspended in a standard suspension vehicle (SSV; 0.9% w/v NaCl, 0.5% w/v Na-carboxymethylcellulose, 0.5% v/v benzyl alcohol, 0.4% v/v Tween-80) and was probe sonicated for 5 min immediately prior to treatment as previously described.^13^

### CET measurements in plasma and brain following acute dose

Six-to-eight week old C57BL/6 male mice (*n* = 3) were administered a single dose of CET (CuATSM: 50 mg/kg, ebselen: 100 mg/kg and telbivudine 150 mg/kg) via oral gavage. Two hours following drug administration mice were delivered an overdose of pentobarbitone and blood samples were collected by puncture of the inferior vena cava. Blood samples were immediately transferred to K3 EDTA tubes and centrifuged to separate plasma, which was snap frozen and stored at −80 °C until analysis. Brains were immediately collected, homogenised in 1 ml phosphate buffered saline (PBS) solution, snap frozen and stored at −80 °C until analysis.

### Plasma and brain selenium level inductively coupled plasma mass spectrometry (ICP-MS) analysis

Selenium (Se) levels were measured in plasma and brain samples as a proxy for ebselen measurements. Frozen homogenised brain (∼200 μL) and plasma samples (15–20 μL) from CET treated mice were freeze dried before being digested for ICP-MS. Briefly, freeze dried samples were weighed and microwave digested with IQ 65% nitric acid (v/v) and Ultrapur 50% hydrogen peroxide (v/v) using the OneTouch method (200 °C max temperature). Following digestion, solutions were dried on a hotplate at 100 °C. Once dried Baseline 0.3 M nitric acid was added and the vials were subsequently sonicated for 15 min. Solutions were then analysed via inductively coupled plasma mass spectrometry (ICP-MS). Digested brain and plasma samples were analysed for Se on a ThermoFisher iCAP-Q ICP-MS at the Wollongong Isotope Geochronology Laboratory. The sample introduction system consisted of a perfluoroalkoxy alkane concentric uFLOW nebuliser, nickel sample and skimmer cones with high matrix insert. Inorganic Ventures IV-ICPMS-71A were used as calibration standard, diluted to concentrations of 0.5, 1, 5, 10, 25, 50 and 100 ppb in Baseline® 0.3 M nitric acid, and IV-ICPMS-71D as the internal standard, diluted to 20 ppb in Baseline® 0.3 M nitric acid. For three samples, two separate aliquots were digested and analysed to assess precision. Precision ranged from 0.8 to 5.6% (1SD) for Se concentrations. Total procedure blanks were determined and were <2 ng for Se. The baseline Se levels of an untreated mouse was subtracted from Se levels obtained from CET treated mice to measure plasma and brain levels of ebselen.

### Subchronic CET toxicity and tolerability study in C57BL/6J mice

Seven week old C57BL/6J mice (*n* = 5 per sex) were administered a daily dose of CET (CuATSM: 50 mg/kg/day, ebselen: 100 mg/kg/day and telbivudine 150 mg/kg/day) via oral gavage for 30 days. Each day during the treatment 15–30 min post-dose mice were observed for any changes in skin and fur, eyes and mucous membranes, respiratory and circulatory function, gait, posture and behaviour, and for the presence of tremors, convulsions or any other abnormal findings. The day after the final treatment mice were fasted for at least 4 h prior to being administered an overdose of pentobarbital and blood samples collected by puncture of the inferior vena cava. Female blood samples were collected in K3-EDTA tubes and stored at room temperature prior to haematology analysis. Male blood samples were collected in serum separator tubes and serum collected and stored at 4 °C prior to biochemical analysis. Haematology and biochemistry measures were performed using an Idexx ProCyte Haematology Analyser and Idexx Catalyst Clinical Chemistry Analyser, as per manufacturer’s instructions.

### Therapeutic efficacy of CET administration in SOD1^G93A^ mice

SOD1^G93A^ mice were randomly assigned to three treatment groups matched for sex, age and litter. Commencing at 50 days of age, 72 SOD1^G93A^ mice (*n* = 12 mice/treatment/sex) were administered either SSV (vehicle), CuATSM (50 mg/kg/day) or CET (CuATSM: 50 mg/kg/day; ebselen: 100 mg/kg/day; telbivudine: 150 mg/kg/day) daily by oral gavage (between 0800 and 1000 h each day). Equivalent volumes of SSV were administered to the vehicle-treated mice. Mice were weighed and scored using the criteria outlined by the ALS Therapy Development Institute^36^ three days a week to assess neurological deficit. Scoring was performed by observers blinded to treatment.

### Mice censured from therapeutic efficacy study trials

In total, four mice (3 CET- and 1 vehicle-treatment groups) were censured from the therapeutic efficacy study for animal welfare issues not related to treatment. Descriptions of all mice censured from trial are outlined in Table 1.

**Table 1:** Description of SOD1^G93A^ mice censured from therapeutic efficacy study.

Treatment	Age (days)	Sex	Reason
CET	108	M	Histopathological showed evidence of gastritis
Vehicle	158	F	Infected eye
CET	172	F	Significant acute weight loss and gait abnormality
CET	191	M	Infected wound from fight with littermate

CET, CuATSM, Ebselen, Telbivudine; M, Male; F, Female.

### Rotarod

To assess the influence of drug treatment on locomotor function, mice were assessed on a rotarod. Two weeks prior to drug treatment mice were habituated to the 5-lane accelerating rotarod (RotaRod Advanced, TSE Systems, Hesse, Germany). Habituation sessions consisted of five acclimatisation sessions, with the first and second session run at an inclining speed of 0–4 rotations per min (rpm) for 180 s. The final three habituation sessions were performed at an inclining speed of 4–20 rpm over a 180 s period. During the recording period (testing) the rotation speed of the rotarod was 4–20 rpm over a 180 s period, with the time taken to fall (latency to fall) recorded for each mouse. Mice were given three independent runs with a 30–60 s rest between runs. The maximum time each mouse was able to remain on the rod was recorded and included in the data analysis. To control for odour cues, the apparatus was cleaned with 70% ethanol after each trial.

### Survival and end-stage

Disease end-stage was defined when a mouse displayed either a 20% loss in maximum body weight and/or reached a clinical score of 4 (inability to right itself within 10 s after being placed on both sides). Once a mouse was identified as reaching end-stage, mice were euthanised via asphyxiation using a slow-fill carbon dioxide technique. Mice were then transcardially perfused with either PBS or 4% paraformaldehyde (PFA), and lumbar spinal cords removed. Tissue collected from mice perfused with PBS was snap frozen in liquid nitrogen and stored at −80 °C. Tissue collected from mice perfused with 4% PFA was post-fixed in ice-cold 4% PFA for 2 h. Following fixation, tissue was rinsed in PBS and cryoprotected in 30% sucrose (w/v), until the tissue sunk. Lumbar regions were then embedded within OCT frozen over liquid nitrogen and stored at −80 °C.

### Lumbar spinal cord homogenisation

PBS perfused lumbar spinal cord (∼30 μg) were homogenised in PBS + 0.1% Triton-X 100 (v/v) and 1 mg/ml N-ethylmaleimide supplemented with Halt™ protease and phosphatase inhibitors (ThermoFisher, Australia, #78444) with polypropylene pestles. Homogenates were centrifuged (14,000×*g* for 30 min at 4 °C) and the supernatant collected. Protein concentration was determined via a DC assay as per the manufacturer’s instructions (Bio-Rad, Australia, #5000111). Tissue homogenate were stored at −80 °C until further analysis.

### Immunoblotting for *in vivo* SOD1 disulfide formation

Lumbar spinal cord homogenates (7.5 μg) mixed with 1:1 with 2 × non-reducing thiol-blocking SDS sample buffer were heated to 70 °C for 10 min prior to being loaded onto stain-free TGX Any-kDa SDS-PAGE precast gels (Bio-Rad, Australia) under non-reducing conditions. Following electrophoresis, total protein on the gel was quantified using a Criterion Stain Fee Imager (Bio-Rad) prior to transferring for immunoblotting. Proteins were transferred onto methanol-activated Amersham Hybond 0.2 μm polyvinylidene difluoride membranes (GE Healthcare, Australia). Membranes were blocked in Tris-buffered saline containing 0.02% Tween 20 (w/v) (TBST) and 5% skim milk powder for 1 h at room temperature. Membranes were then incubated with anti-SOD1 antibody (Abcam, ab13498; RRID:AB_300402, 1:10,000) in blocking solution overnight. Following primary antibody incubation, membranes were washed three times in TBST and then incubated with horseradish peroxidase-conjugated anti-rabbit IgG (Dako, P0448; RRID:AB_2617138, 1:5000) in blocking solution containing 2.5% skim milk for 1 h at room temperature. To visualise bands, membranes were incubated with enhanced chemiluminescent reagent (GE Healthcare, Australia) and imaged with an Amersham 600RB imager (GE Healthcare). SOD1 band quantification (S–S and S–H) was achieved through the use of ImageJ software (version 1.53c, National Institutes of Health). Sample intensity was normalised to total protein and a pooled sample (containing equal amounts of each sample) to account for equal protein loading between samples and gels, respectively.

### In-gel zymography to measure SOD1 activity *in vivo*

To determine SOD1 activity, 2.5 μg of lumbar spinal cord homogenates and 100 ng of purified recombinant human SOD1^WT^ protein were separated in 8% (v/v) native PAGE gels. Following electrophoresis, gels were incubated in 5 mM nitrotetrazolium blue chloride (Sigma–Aldrich, #N5514) for 20 min with gentle agitation, before incubation in developer solution (10 mM tetramethylethylenediamine and 30 μM riboflavin) for 15 min. Subsequently, gels were exposed to fluorescent light until sufficient contrast between the achromatic zones and background was obtained. Gels were then imaged on a calibrated densitometer (GS-900; Bio-Rad, Australia). The activity of SOD1 was quantified as the intensity of bands using Image J software (version 1.53c, National Institutes of Health) and normalised to total SOD1 levels previously determined via immunoblot.

### Dot blot

Lumbar spinal cord homogenate aliquots were prepared at equal concentrations and volumes in PBS containing 0.1% Triton-X 100. Homogenates (2.5 μg) were deposited onto nitrocellulose membranes and samples were allowed to dry at room temperature. Membranes were blocked in 5% skim milk powder/TBST (w/v) for 1 h, followed by overnight incubation with misfolded SOD1-specific C4F6 antibody (MédiMabs, #MM-0070-2-P; RRID:AB_10015296, 1:2500). After overnight incubation, membranes were washed three times in TBST before addition of horseradish peroxide-conjugated secondary antibody for anti-rabbit IgG (Dako, P0448; RRID:AB_2617138, 1:5000) in blocking solution containing 5% skim milk (w/v) for 1 h. To visualise signal, membranes were incubated with ECL (GE Healthcare, Australia) and imaged with an Amersham 600RB imager (GE Healthcare). To ensure equal protein loading, membranes were washed as above, incubated with Ponceau S stain solution for 5 min, rinsed with distilled water and then imaged. Quantification of C4F6 and Ponceau S signal was achieved through the use of ImageJ software (version 1.53c, National Institutes of Health). C4F6 signal intensity was normalised to Ponceau S for each sample to account for protein loading.

### Histology and motor neuron counts

PFA-perfused lumbar spinal cord tissue was cryosectioned (12 μm) with 1:10 series thaw mounted on PolylysineTM slides (Sigma–Aldrich, Castle Hill, NSW, Australia). Slides were washed three x 5 min in phosphate buffered saline. Motor neuron number within the lumbar spinal cord was assessed as previously described, with minor modifications.^37^ Briefly, sections were incubated in 0.1% (w/v) thionin acetate stain for 1 min. Following staining, slides were gently rinsed with running tap water for 30 s to remove excess stain. Microscope examination at this stage ensured adequate staining intensity. Slides were thereafter left to air-dry overnight in a fume hood to ensure complete drying prior to mounting with DPX mountant (Sigma–Aldrich, Castle Hill, NSW, Australia). Images of stained spinal cords were acquired using the Leica DM750 microscope on a DM750 (Leica, Wetzlar, Germany).

Motor neurons from stained lumbar spinal cord slice images were counted using a user-assisted automated process. CellProfiler software (version 4.2.4 for Windows, Broad Institute, Cambridge, Massachusetts) was firstly used to identify and outline cells with an area equivalent to a 20 μm diameter or greater. Briefly, the *Unmix Colours* module was used to generate greyscale output images, based on the unique absorbance of the thionin acetate stain. Illumination correction of these images was then performed using the *Smooth* and *ImageMath* modules, as described above. Following illumination correction, the *Identify Primary Objects* module, with a Global Otsu Two Class threshold strategy, was used to identify cells with an equivalent diameter greater than 20 μm. The *Overlay Outlines* module then generated images with the identified “objects” overlayed onto the original images. Motor neuron numbers were identified by counting outlined cells in the ventral horn, excluding any outlined artefacts, large cells identified outside the ventral horn region, or outlined clumps of cells that had not been properly segmented. Counts were performed by two blind observers, and the counts averaged. Motor neuron counts presented per mouse represent the average number of motor neurons from 4 to 8 separate sections.

### Statistics

All statistical analysis was performed using GraphPad Prism software version 9.5.1 for Windows. Normality of data was assessed by a Shapiro–Wilk test, prior to performing comparative statistical analysis. For One-way ANOVA and repeated one-way ANOVA homoscedasticity and sphericity of the data were determined by a Brown-Forsythe and Mauchly’s Test of Sphericity. One-way ANOVA with Tukey’s multiple comparisons post-hoc analysis performed to statistically analyse cell based experiments. Longitudinal body weight, neurological score and rotarod data were analysed through repeated measures one-way ANOVA with Tukey’s post-hoc tests for comparisons between vehicle (control), CuATSM and CET treatment groups. Age of peak body weight and disease onset were statistically analysed by one-way ANOVA with Tukey’s multiple comparisons post-hoc analysis. Survival is shown as Kaplein–Meier plots and median survival analysed using one-sample Wilcoxon test to compare treatment groups. A one-way ANOVA with Tukey’s post-hoc tests were used to statistically analyse total, disulfide bonded and activity levels of SOD1, in addition to motor neuron counts. All data is represented as mean ± SEM, unless indicated and significance was set at an alpha level of p < 0.05.

## Results

### CET combination therapy is more effective than CuATSM in protecting against SOD1^G93A^ pathology in cultured cells

**Fig. 1:**
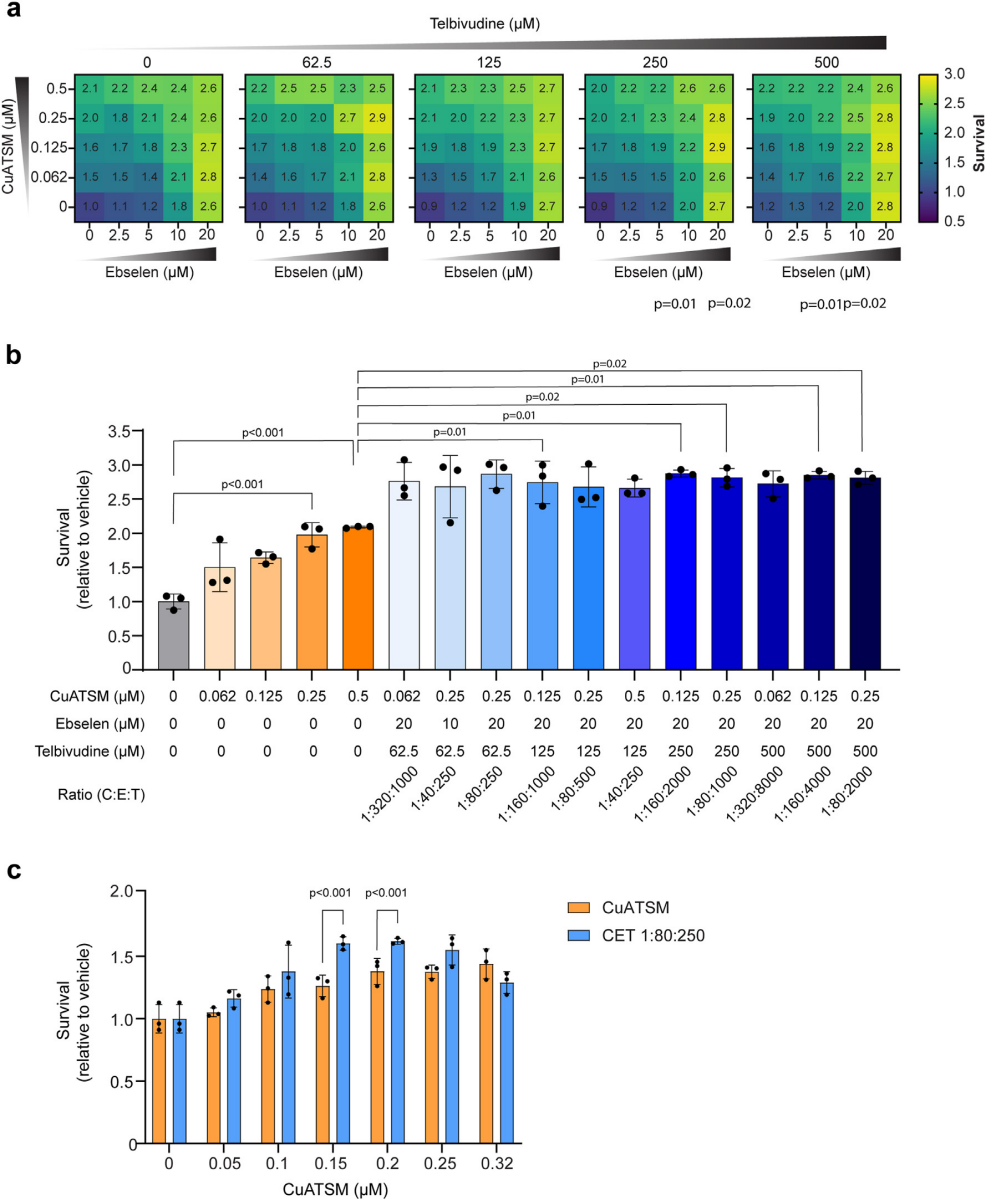
**Establishing the effect of CET combination therapy on cell survival of NSC-34 cells expressing SOD1^G93A^-EGFP. (a)** Heatmaps of the 3D checkerboard treatment of NSC-34 cells expressing SOD1^G93A^-EGFP. The colour scale indicates the degree to which treatment improves survival, and numbers indicate the fold increase in survival relative to the vehicle control. **(b)** The CET ratios that resulted in the greatest improvements in SOD1^G93A^-EGFP cell survival (≥2.7 fold) from **(a)** were compared against CuATSM treatment alone. **(c)** A ratio of 1:80:250 for the CET combination therapy was selected to move forward for further testing using a range of CuATSM concentrations in NSC-34 cells expressing SOD1^G93A^-EGFP. The CET combination therapy with a CuATSM concentration of 0.15 μM and 0.2 μM improved the survival of SOD1^G93A^-expressing cells compared to the equivalent concentration of CuATSM alone (determined by a repeated one-way ANOVA with Tukey’s multiple comparisons post-test. All data represent mean ± SD (*n* = 3 individual experiments).

Based on previous evidence that CuATSM, ebselen and telbivudine reduce SOD1 toxicity, we aimed to establish whether a combination of CuATSM, ebselen and telbivudine would be more effective than CuATSM in protecting against SOD1-associated ALS pathology. To establish the optimal ratio of drug combination to be used in a cell culture model of SOD1-associated ALS, a 3D checkerboard assay was employed to test varying concentrations of CuATSM (0–0.5 μM), ebselen (0–20 μM) and telbivudine (0–500 μM on the survival of neuronal-like NSC-34 cells expressing SOD1^G93A^-EGFP (Fig. 1a and Supplementary Table S1). CuATSM treatment increased cell survival in a dose-dependent manner (Fig. 1a and b). The CuATSM, ebselen and telbivudine (CET) treatments that improved survival to the greatest extent (≥2.7 fold relative to the vehicle control) were then compared against all concentrations of CuATSM alone tested (Fig. 1b and Supplementary Fig. S1). All CET treatments with the exception of one improved cell survival compared to the equivalent concentration of CuATSM alone. Furthermore, multiple ratios of the CET treatment (1:80:250, 1:160:2000, 1:80:1000, 1:160:4000, 1:80:2000; CuATSM:ebselen:telbivudine) comprising of a concentration of CuATSM ≤ 0.25 μM, were found to improve survival compared to the highest concentration of CuATSM alone tested (0.5 μM). As the 1:80:250 CET ratio improved survival to the highest level and to equivalent and higher concentrations of CuATSM, it was selected for further testing. We next tested the 1:80:250 CET ratio against a broad range of CuATSM concentrations (0–0.32 μM) and imaged SOD1^G93A^-EGFP transfected cells every 4 h for a 48 h period to monitor cell survival. 0.32 μM was selected as the highest concentration of CuATSM tested, as this was previously determined as the peak therapeutic concentration for SOD1^G93A^-EGFP expressing cells.^38^ Using CuATSM concentration of 0.15 μM or 0.20 μM in the 1:80:250 CET ratio improved cell survival compared to equivalent concentration of CuATSM alone (Fig. 1c). The level of cell survival observed in the experiments shown in Fig. 1c were not as high as observed in the initial checkerboard assays (Fig. 1a–b), likely due to the repeated fluorescent imaging of the cells and potentially causing free radical accumulation.^39^ However, consistent with previous experiments, we continued to observe the trend that CET improved survival compared to CuATSM alone. The 1:80:250 CET ratio with concentrations of 0.15 μM CuATSM, 12 μM ebselen, 37.5 μM telbivudine was therefore selected to move forward for further testing.

CET combination therapy is more effective than CuATSM treatment in increasing the survival of cells expressing SOD1^G93A^ and reducing SOD1^G93A^ inclusion formation.

**Fig. 2:**
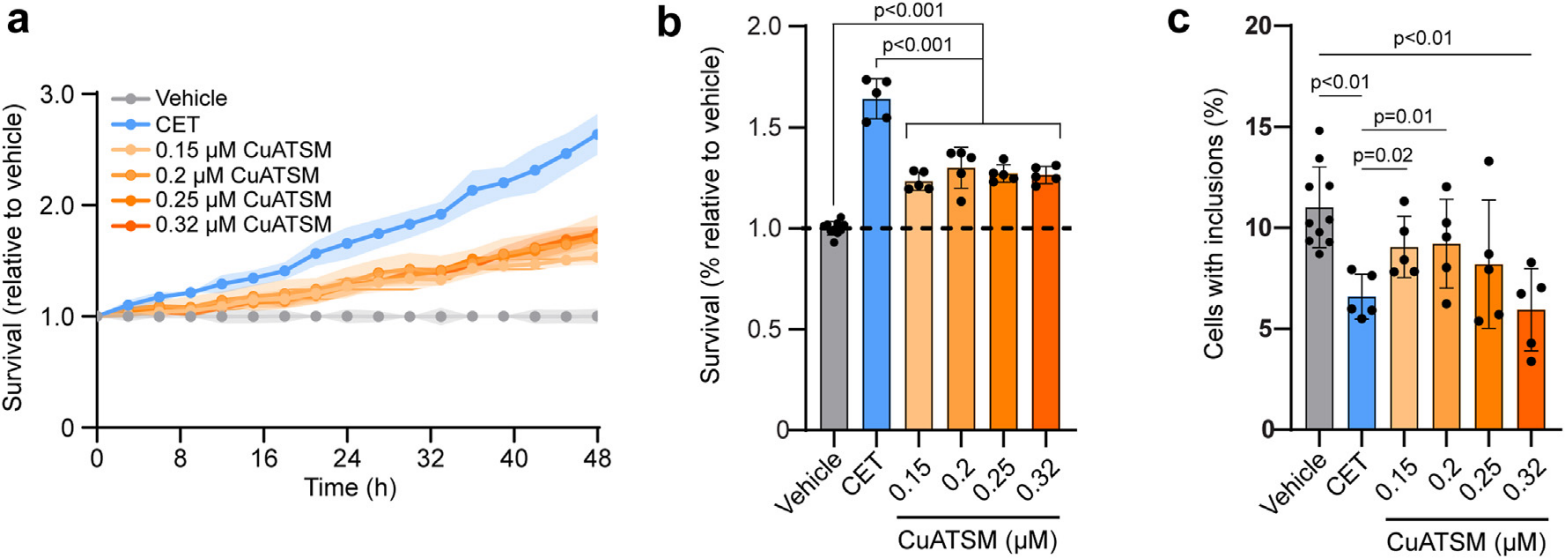
**CET combination therapy is more effective than CuATSM treatment in increasing the survival of cells expressing SOD1^G93A^ and reducing SOD1^G93A^ inclusion formation. (a)** NSC-34 cells expressing SOD1^G93A^-EGFP were treated with either CuATSM (0.15 μM), ebselen (12 μM) and telbivudine (37.5 μM) (CET) combination therapy or CuATSM alone and imaged every 3 h for 48 h on an IncuCyte S3 automated fluorescent microscope and GFP cell counts quantified. **(b)** The survival of SOD1^G93A^-EGFP expressing cells was determined by measuring the area under the GFP count curves **(a)**. Solid lines represent mean values with shaded areas representing ± SD (n ≥ 5 technical replicates from at least 3 separate experiments) One-way ANOVA with a Tukey’s multiple comparisons post-test was used to compare statistical significance between treatments. **(c)** The number of cells containing SOD1^G93A^-EGFP inclusions was determined by semi-automated analysis of the 48 h time point images from the IncuCyte survival assay. One-way ANOVA with a Tukey’s multiple comparisons post-test was used to compare statistical significance between treatments.

Having established an appropriate ratio and concentrations of CuATSM (0.15 μM), ebselen (12 μM) and telbivudine (37.5 μM) to use for the CET polytherapy approach in our cell model, we sought to compare the effects of CET administration on cell survival and SOD1 inclusion formation compared to CuATSM treatment alone (0.15 μM–0.32 μM). Similar to what has been observed previously,^20^^,^^38^ CuATSM was found to improve the survival of NSC-34 cells expressing SOD1^G93A^-EGFP compared to the vehicle control (Fig. 2a and b). Moreover, CET polytherapy improved the survival of NSC-34 cells expressing SOD1^G93A^-EGFP compared to all concentrations of CuATSM tested (Fig. 2a and b). CuATSM treatment has also been shown to decrease SOD1 inclusion formation.^19^^,^^20^ In accordance with these previous studies, the percentage of cells containing SOD1^G93A^-EGFP inclusions was reduced by CuATSM treatment at 0.32 μM and CET combination therapy compared to vehicle treated cells (Fig. 2c and Supplementary Fig. S2). CET polytherapy was also more effective at decreasing inclusion formation in cells than both 0.15 μM and 0.2 μM CuATSM alone (Fig. 2c). Moreover, CET polytherapy showed a similar reduction in inclusion formation to the highest concentration of CuATSM tested (0.32 μM) despite it having less than half the concentration of CuATSM.

### CET combination therapy improves SOD1^G93A^ folding in NSC-34 cells

**Fig. 3:**
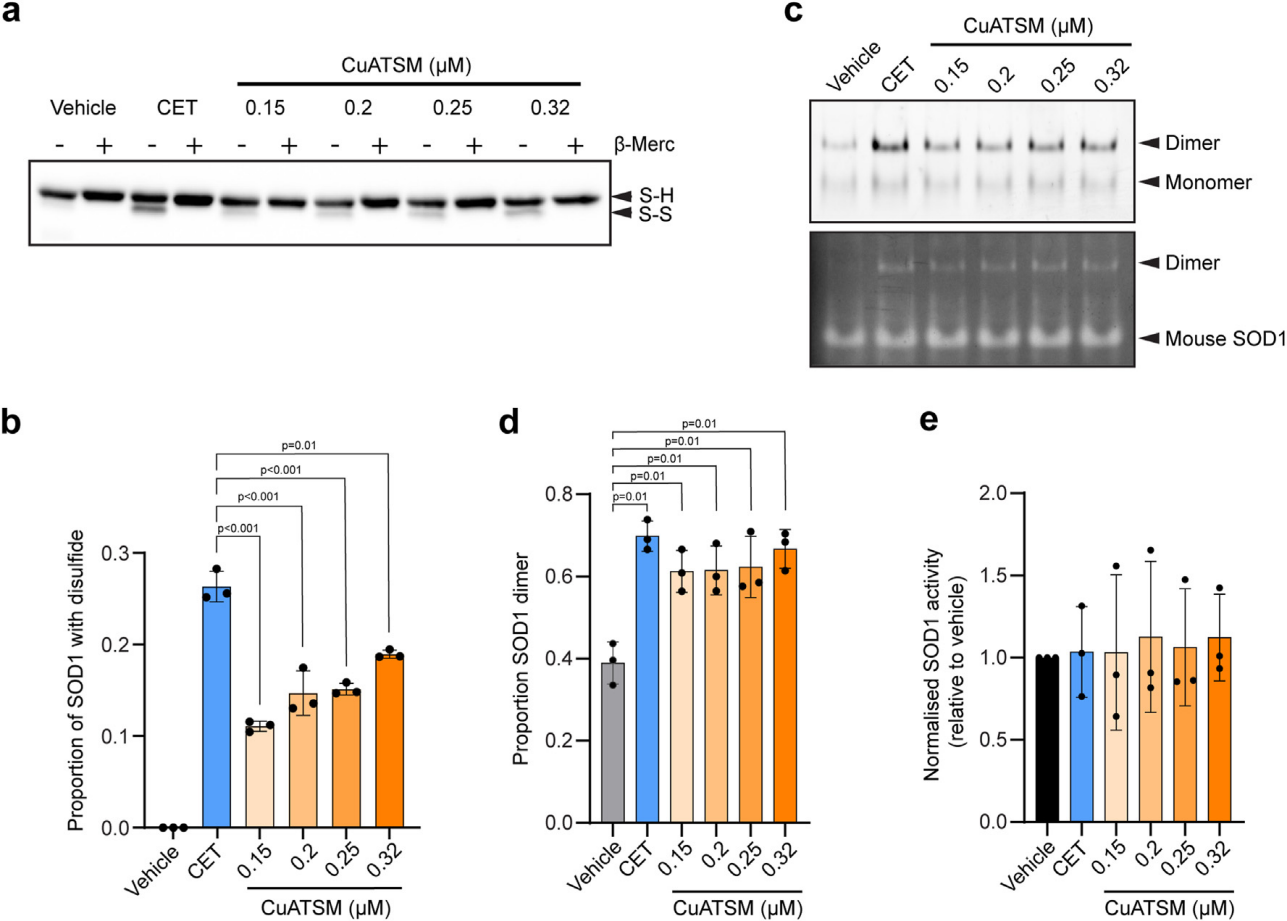
**CET polytherapy improves SOD1^G93A^ folding but does not alter SOD1 activity in NSC-34 cells. (a)** The proportion of disulfide-bonded SOD1 was determined by SDS-PAGE migration of SOD1^G93A^-EGFP lysates treated with either CET or CuATSM under reducing conditions (+β-merc) and nonreducing conditions (−β-merc). Arrows indicate the position of disulfide-bonded SOD1 (SS) and reduced SOD1 (SH) on the immunoblot. **(b)** Quantification of the immunoblots from **(a)** by densitometry. Data shown are mean ± SD (n = 3) and statistical significance was determined by one-way ANOVA with a Tukey’s multiple comparisons post-test. **(c)** Native-PAGE (top) and in-gel zymography (bottom) of SOD1^G93A^-tdTomato cell lysates treated with either CET or CuATSM. The position of SOD1 dimers, monomers and endogenous mouse SOD1 are indicated with arrows. **(d)** Quantification of the tdTomato signal from native-PAGE to determine the proportion of SOD1 dimer present following CET or CuATSM treatment. Data shown are mean ± SD (n = 3) and statistical significance was determined by one-way ANOVA with a Tukey’s multiple comparisons post-test. Asterisks indicate significant difference to the vehicle control. **(e)** SOD1 activity (normalised to SOD1 protein levels) was measured in cells treated with either CET or CuATSM by quantifying the achromatic bands corresponding to the SOD1^G93A^ dimer from in-gel zymography and normalising to the corresponding tdTomato fluorescence signal from the native-PAGE gel. Data represent mean ± SD (n = 3). One-way ANOVA was used to determine significant differences in SOD1 activity between treatments.

To investigate whether CET combination therapy was more effective than CuATSM treatment at promoting SOD1 maturation, we next assessed the intramolecular disulfide bond formation and activity of SOD1^G93A^. Non-reducing SDS-PAGE has been shown to maintain the intramolecular disulfide bond in SOD1, with this species of SOD1 migrating faster in-gel electrophoresis than reduced SOD1.^19^^,^^40^ Following 48 h treatment with either CET or CuATSM, intramolecular disulfide-bonded SOD1 was detected by immunoblot (Fig. 3a). CET treatment increased the proportion of intramolecular disulfide-bonded SOD1 compared to CuATSM, even when the concentration of CuATSM was more than two-fold that used in the CET treatment (Fig. 3b).

Considering formation of the intramolecular disulfide bond in SOD1 promotes dimer formation and increases the activity of the SOD1 enzyme,^40^ we next sought to determine the effects of CET combination therapy on SOD1 enzymatic activity. For this set of experiments, SOD1^G93A^ with a tdTomato tag was used, as this allowed for greater separation between different SOD1 species by native-PAGE from endogenous SOD1. Imaging of the tdTomato signal following native-PAGE highlighted that both CET combination therapy and CuATSM treatment increased the proportion of SOD1 dimer relative to the vehicle control (Fig. 3c and d), however CET and CuATSM-treated cells exhibited similar proportions of SOD1 dimer. In line with this, CET and CuATSM treatment displayed akin SOD1 activity levels (Fig. 3c and e), indicating that the treatment-induced increases in SOD1 protein involved increased abundance of SOD1 in its mature, physiologically active form.

### CET is bioavailable in the CNS

Prior to commencing treatment of CET in an ALS-associated mouse model, an acute dosage study was performed to evaluate the bioavailability of all three small molecules. C57BL/6 mice were administered a single oral dose of CET treatment (50 mg/kg CuATSM, 100 mg/kg ebselen and 150 mg/kg telbivudine) and plasma and brain tissue collected. Selenium levels have shown to closely correlate with plasma levels of ebselen and its metabolites levels.^41^^,^^42^ Therefore, selenium levels were used as a proxy measure of ebselen levels. Measurable levels of CuATSM, ebselen (selenium) and telbivudine were obtained in the plasma and brain 2 h following a single oral administration of CET (Table 2).

**Table 2:** *In vivo* concentration of CuATSM, selenium (ebselen) and telbivudine in C57BL/6 mice 2 h following a single oral dose of CET (50 mg/kg CuATSM, 100 mg/kg ebselen and 150 mg/kg telbivudine).

	CuATSM	Selenium (ebselen)	Telbivudine
Plasma	82.13 ± 19.72 ng/ml	17.72 ± 3.15 μg/ml	15.50 ± 3.14 μg/ml
Brain	189.87 ± 31.66 ng/g	1.84 ± 3.44 μg/g	0.94 ± 0.01 μg/g
Brain:Plasma ratio	2.4 ± 0.3	0.10 ± 0.01	0.06 ± 0.00

Results are presented as mean ± SD. n = 3 mice.

### Subchronic oral gavage administration of CuATSM, ebselen and telbivudine in C57BL/6 is tolerable and does not induce adverse events

We have previously observed that relatively high doses of CuATSM (100 mg/kg/day) can result in adverse events in a subset of SOD1^G93A^ mice maintained on a C57BL/6J background strain.^13^ To ensure CET tolerability, C57BL/6J were orally gavaged CET (50 mg/kg CuATSM, 100 mg/kg ebselen and 150 mg/kg telbivudine) for 30 consecutive days. Daily weight measurements showed the body weight of both male and female mice increased 1.33 ± 0.09 g (5.44%) and 0.68 ± 0.04 g (3.60%) compared to their pre-treatment weight following 30 days of daily CET administration. Daily clinical assessment of CET-treated mice showed no evidence of tremors, convulsions or any other abnormal findings, with no changes to skin and fur, eyes and mucous membranes, respiratory and circulatory function, gait and posture, or behaviour. In addition, terminal haematology and serum biochemistry following 30 days of CET administration showed negligible changes across a panel of markers (Table 3, Table 4), indicating the combination of these compounds at these doses was safe and tolerable for a long-term therapeutic efficacy study.

**Table 3:** Haematology results of female C57BL/6J mice following 30 days of oral CET (50 mg/kg CuATSM, 100 mg/kg ebselen, 150 mg/kg telbivudine) administration.

	Units^b^	Control^b^	CET^b^	Reference data^c^
Red blood cells	x10ˆ12/L	9.5 ± 0.5	8.8 ± 0.8	
Haematocrit	L/L	44.5 ± 1.9	39.9 ± 4.6	
Haemoglobin	g/dL	14.4 ± 0.8	**12.9 ± 1.4**^a^	12.0 ± 0.6
MCV	fL	46.5 ± 0.9	45.2 ± 1.5	
MCH	pg	15.0 ± 0.0	**14.6 ± 0.4**^a^	14.0 ± 0.8
MCHC	g/dL	32.4 ± 0.8	32.2 ± 0.5	
% Reticulocyte	%	4.1 ± 0.6	4.1 ± 1.3	
Reticulocytes	1000/μL	394 ± 55	355 ± 80	
White blood cells	x10ˆ9/L	4.3 ± 1.5	3.1 ± 0.5	
Neutrophils %	%	16.2 ± 11.7	24.7 ± 11.7	
Lymphocytes %	%	78.6 ± 10.2	70.8 ± 11.3	
Monocytes %	%	3.1 ± 2.4	1.9 ± 1.0	
Eosinophils %	%	*2.1 ± 1.4*	*2.5 ± 1.3*	
Basophils %	%	*0.0 ± 0.0*	*0.1 ± 0.2*	
Neutrophils	x10ˆ9/L	*0.7 ± 0.4*	*0.8 ± 0.4*	
Lymphocytes	x10ˆ9/L	3.4 ± 1.4	2.2 ± 0.6	
Monocytes	x10ˆ9/L	*0.1 ± 0.1*	***0 ± 0***^a^	*0.26 ± 0.17*
Eosinophils	x10ˆ9/L	*0.1 ± 0.1*	***0 ± 0***^a^	*0.09 ± 0.09*
Basophils	x10ˆ9/L	0.0 ± 0.0	0 ± 0	
Platelets	x1000/μL	*1098 ± 464*	*601 ± 321*	

Results are presented as mean ± SD.

MCV, Mean Cell Volume; MCH, Mean Cell Haemoglobin; MCHC, Mean Cell Haemoglobin Concentration.

a Bolded values indicate p < 0.05 compared to control. Italicized values indicate data were skewed. n = 17–18 for control group and n = 4–5 for combination therapy group.

b Control group data was collected from a previous study, whereby mice where orally gavaged with saline.

c The Jackson Laboratory, Physiological data summary of 24 week old C57BL/6J female mice.

**Table 4:** Serum biochemistry of male C57BL/6J mice following 30 days of oral CET (50 mg/kg CuATSM, 100 mg/kg ebselen, 150 mg/kg telbivudine) administration.

	Units^b^	Control^b^	CET	Reference data^43^
Glucose	mmol/L	15.2 ± 3.7	14.5 ± 1.3	
Urea	mmol/L	7.7 ± 0.9	8.4 ± 1.0	
Phosphorus	mmol/L	2.8 ± 0.3	2.8 ± 0.2	
Total Protein	g/L	46.8 ± 2.6	45.0 ± 1.7	
Albumin	g/L	25.9 ± 2.0	27.6 ± 0.6	
Globulin	g/L	21.2 ± 3.6	17.4 ± 1.5	
Albumin:Globulin		1.3 ± 0.3	1.6 ± 0.1	
AST	U/L	58.6 ± 34.6	40.8 ± 14.0	
ALP	U/L	190 ± 58	**112 ± 11**^a^	68–132
Cholesterol	mmol/L	2.1 ± 0.5	1.9 ± 0.5	
Creatine Kinase	U/L	*262 ± 354*	*246 ± 321*	
Creatinine	μmol/L	11.0 ± 3.0	11 ± 2.7	
Calcium	mmol/L	2.4 ± 0.1	2.1 ± 0.2	
ALT	U/L	37.2 ± 45.1	26.3 ± 2.9	
Bilirubin - Total	μmol/L	4.0 ± 1.1	3.5 ± 0.6	
Triglyceride	mmol/L	*0.8 ± 1.1*	*1.3 ± 0.3*	
Sodium	mmol/L	151 ± 3.5	152 ± 2.5	
Potassium	mmol/L	5.4 ± 1.0	6.4 ± 0.5	
Chloride	mmol/L	107 ± 2.0	**112 ± 1.6**^a^	104–114

Results are presented as mean ± SD.

AST, Aspartate aminotransferase; ALP, Alkaline phosphatase; ALT, Alanine aminotransferase.

a Bolded values indicate p < 0.05 compared to control. Italicized values indicate skewed data. n = 17–18 for control group and n = 4–5 for combination therapy group.

b Control group data was collected from a previous study, whereby mice where orally gavaged with saline.

### CET treatment delays disease onset and increases survival in SOD1^G93A^ mice

**Fig. 4:**
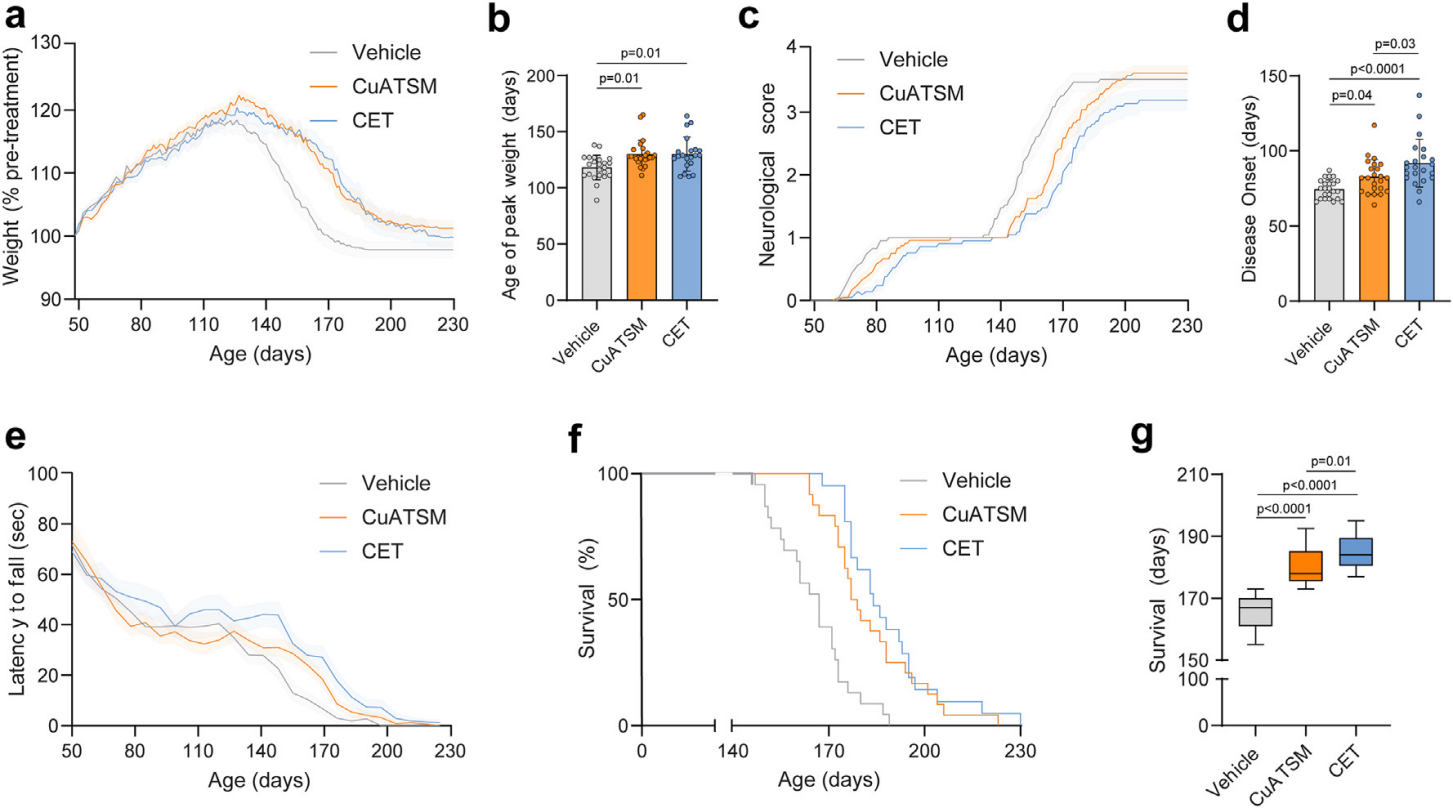
**CET polytherapy delays disease onset, modifies neurological scoring and extends survival in SOD1^G93A^ mice.** The effect of daily oral gavage administration of either vehicle, CuATSM or CET on **(a)** body weight, **(b)** age of peak body weight, **(c)** neurological score, **(d)** age of disease onset (defined as attaining a neurological score of 1), **(e)** latency to fall during rotarod task, **(f)** Kaplain–Meier plot and **(g)** survival **(a, c and e)** Solid lines represent mean values with shaded areas representing ± SEM (*n* = 21–24 per treatment group). **(b and d)** Data is shown as mean ± SD (*n* = 20–24 per treatment group). **(g)** Data is shown as median ± IQR.

Given the promising *in vitro* efficacy and *in vivo* safety/distribution results, we next sought to determine whether CET treatment exhibits therapeutic benefits when administered to the transgenic SOD1^G93A^ mouse model of ALS. SOD1^G93A^ mice were orally gavaged with either standard vehicle suspension, CuATSM (50 mg/kg/day) or CET (50 mg/kg/day CuATSM, 100 mg/kg/day ebselen and 150 mg/kg/day telbivudine) daily commencing at 50 days old (pre-symptomatic). SOD1^G93A^ mice were weighed every three days from 50 days of age. A repeated measures one-way ANOVA of body weight (reported as % of pre-treatment) throughout treatment duration revealed CuATSM- and CET-treated mice displayed an increased mean body weight (% of pre-treatment) than vehicle-treated mice throughout the duration of the study (CuATSM: mean difference (MD) (95% CI): 4.25 (3.72–4.98), p < 0.0001, repeated one-way ANOVA with Tukey’s multiple comparisons post-hoc CET: MD (95% CI): 3.72 (2.94–4.51), p < 0.0001, repeated one-way ANOVA with Tukey’s multiple comparisons post-hoc) (Fig. 4a). Whilst all treatment groups displayed similar maximum body weights (% of pre-treatment), both CuATSM- and CET-treated mice reached maximum body weight later than vehicle-treated mice (CuATSM: mean difference (MD) (95% CI): 11.87 (2.88–20.86), p = 0.01; CET: MD (95% CI): 11.93 (2.63–21.23), p = 0.01, one-way ANOVA with Tukey’s multiple comparisons post-hoc) (Fig. 4b).

The onset and progression of disease were assessed using the standard ALS neurological scoring criteria (Fig. 4c).^36^ The age at which symptoms were first observed (hindleg clasping) was delayed in CuATSM-treated mice compared to vehicle-treated mice (MD (95% CI): 8.30 (0.08–16.69), p = 0.04, one-way ANOVA with Tukey’s multiple comparisons post-hoc). CET-treatment delayed disease onset by 23% and 10% compared to vehicle- (MD (95% CI): 17.39 days (8.91–25.86), p < 0.0001, one-way ANOVA with Tukey’s multiple comparisons post-hoc) and CuATSM-treated mice (MD (95% CI): 9.1 days (0.60–17.56), p = 0.03, one-way ANOVA with Tukey’s multiple comparisons post-hoc), respectively (Fig. 4d). Sex-specific analysis showed both male- and female-treated CET-treated mice delayed disease onset compared to vehicle-treated mice (males: MD (95% CI): 20.08 (6.84–33.33), p = 0.01, one-way ANOVA with Tukey’s multiple comparisons post-hoc); females: MD (95% CI): 16.36 (4.98–27.74), p = 0.01, one-way ANOVA with Tukey’s multiple comparisons post-hoc) (Supplementary Fig. S3a and d). Longitudinal analysis of neurological score showed that CuATSM-treated mice exhibited a lower neurological score than vehicle-treated mice (MD (95% CI): −0.23 (−0.29 to −0.16), p < 0.0001, repeated one-way ANOVA with Tukey’s multiple comparisons post-hoc). Meanwhile, CET-treated mice exhibited a lower neurological score than both vehicle- (MD (95% CI): −0.51 (−0.58 to −0.44), p < 0.0001, repeated one-way ANOVA with Tukey’s multiple comparisons post-hoc) and CuATSM-treated mice (MD (95% CI): 0.28 (−0.31 to −0.25), p < 0.0001, repeated one-way ANOVA with Tukey’s multiple comparisons post-hoc).

To examine motor function and co-ordination, SOD1^G93A^ mice completed a weekly rotarod task and the latency to fall recorded. Whilst vehicle- and CuATSM-treated mice displayed similar latencies to fall, CET-treated mice showed an extended latency to fall compared to both vehicle- (MD (95% CI): 7.84 (4.44–11.23), p = 0.04, repeated one-way ANOVA with Tukey’s multiple comparisons post-hoc) and CuATSM-treated mice (MD (95% CI): 5.91 (3.36–8.46), p = 0.04, repeated one-way ANOVA with Tukey’s multiple comparisons post-hoc) (Fig. 4e).

The age SOD1^G93A^ mice reached humane end-point was assessed when mice were unable to right themselves within 10 s after being placed on either side or a 20% loss in maximum body weight. Analysis of median survival revealed that CuATSM-treated mice exhibited 7% increase compared to vehicle-treated mice (median difference (95% CI): 11.00 (8.00–25.00), p < 0.0001, one-sample Wilcoxon test) (Fig. 4f–g). CET-treated mice exhibited a 10% increase in median survival age compared with vehicle-treated mice (median difference (95% CI): 17.00 (14.00–29.00), p < 0.0001, one-sample Wilcoxon test) and a 3% increase compared to CuATSM-treated mice (median difference (95% CI): 6.00 (2.00–13.00), p = 0.01, one-sample Wilcoxon test). Sex-specific analysis revealed only female-treated CET mice exhibited an increase in survival compared to CuATSM-treated mice (median difference (95% CI): 15.50 (1.00–21.00), p = 0.02, one-sample Wilcoxon test) (Supplementary Fig. S3e and f), whilst male CuATSM- and CET-treated mice displayed similar survival ages (median difference (95% CI): −3.00 (−18.00 to 13.00), p = 0.74, one-sample Wilcoxon test) (Supplementary Fig. S3b and c).

### CET administration improves SOD1 maturation and reduces misfolded SOD1 accumulation *in vivo*

**Fig. 5:**
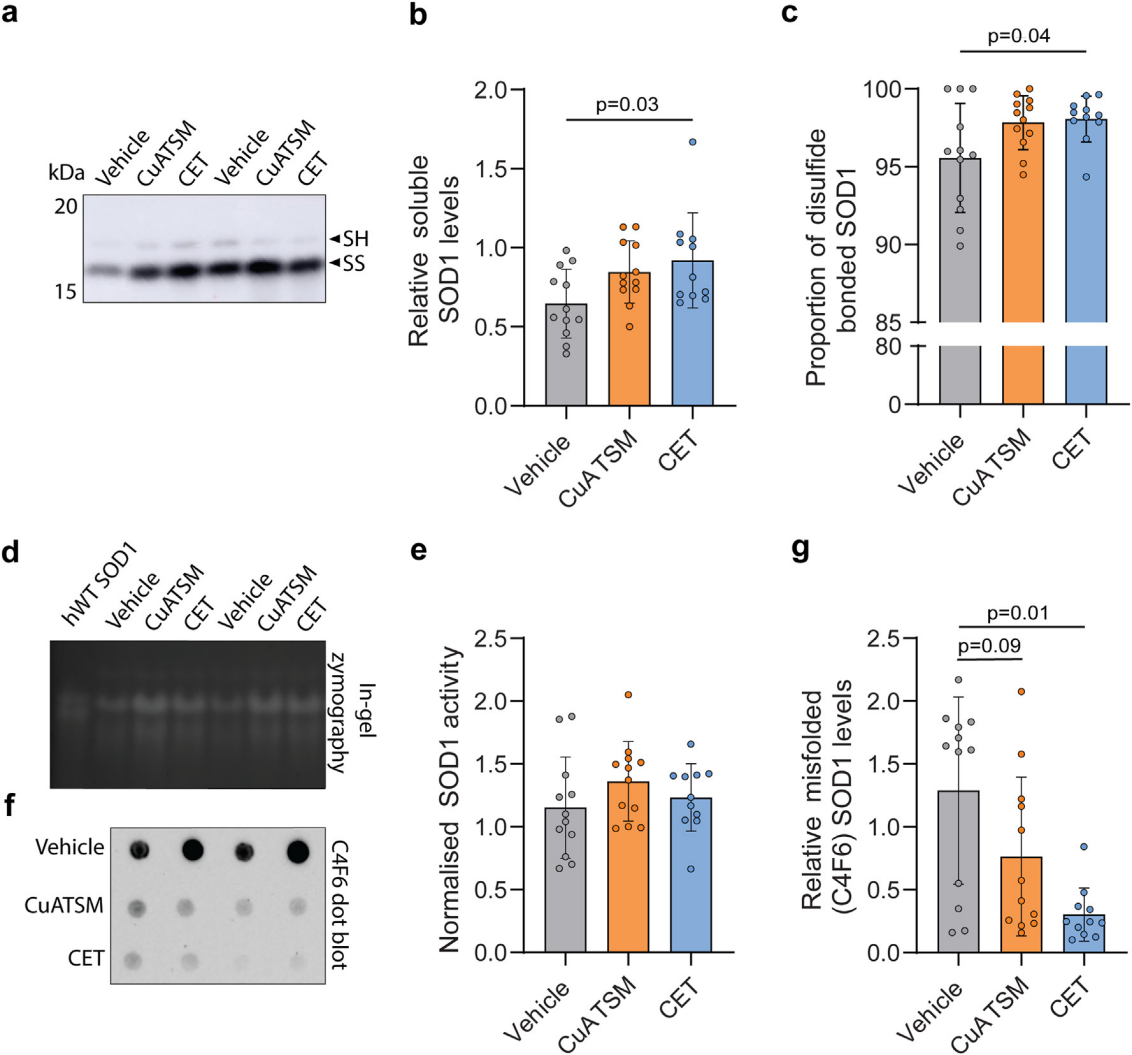
**CET treatments increases *in vivo* SOD1 maturation and reduces misfolded SOD1 accumulation in SOD1^G93A^ mice. (a and b)** The relative levels of SOD1 protein and **(c)** the proportion of disulfide SOD1 (reduced = SH, oxidised intact = SS) in lumbar spinal cord homogenates of vehicle-, CuATSM- and CET-treated mice as determined by non-reducing immunoblot. **(d)** SOD1 in-gel zymography and **(e)** quantification of SOD1 activity normalised to soluble SOD1 levels in lumbar spinal cord homogenates of vehicle-, CuATSM- and CET-treated mice. **(f)** C4F6 dot blots of lumbar spinal cord homogenates and **(g)** relative misfolded SOD1 (C4F6) levels in lumbar spinal cord homogenates of vehicle-, CuATSM- and CET-treated mice. Data shown are mean ± SD (*n* = 11–12/treatment). Data are from 3 technical replicates. One-way ANOVA was used to compare relative differences between vehicle-, CuATSM- and CET-treated SOD1^G93A^ mice.

To investigate the impact of treatment with either CuATSM or CET on SOD1 levels *in vivo*, PBS-soluble fractions were prepared from the lumbar spinal cords of CuATSM-, CET- and vehicle-treated mice. Immunoblots for SOD1 under non-reducing SDS-PAGE conditions were performed to enable assessment of total soluble SOD1 protein levels and the proportion of intramolecular disulfide bonded SOD1 (Fig. 5a).^40^ CET-treated mice exhibited 42.1% higher levels of PBS-soluble SOD1 compared to vehicle-treated mice (Fig. 5b). Furthermore, CET-treated mice exhibited a 2.6% increased proportion of intramolecular disulfide bonded SOD1 compared to vehicle-treated mice (Fig. 5c), indicating a higher degree of SOD1 maturation.

To assess if SOD1 maturation was associated with increased SOD1 activity in-gel zymography was performed (Fig. 5d). CuATSM- and CET-treated mice exhibited overall higher total SOD1 activity compared to vehicle-treated mice, however activity levels were negligible when normalising the activity of each sample to total SOD1 protein levels (Fig. 5e). Lastly, we performed dot-blots on lumbar PBS-soluble fractions using the C4F6 antibody to detect levels of misfolded SOD1 (Fig. 5f).^44^^,^^45^ CuATSM -treated mice showed a trend towards a reduced amount of misfolded SOD1 compared to vehicle-treated mice (Fig. 5g). However, CET-treated mice exhibited a larger reduction in misfolded SOD1 compared to vehicle-treated mice (76.5%), indicating a reduced accumulation of misfolded SOD1 in the lumbar spinal cord of the CET-treated SOD1^G93A^ mice.

### CuATSM and CET administration provides neuroprotection *in vivo*

**Fig. 6:**
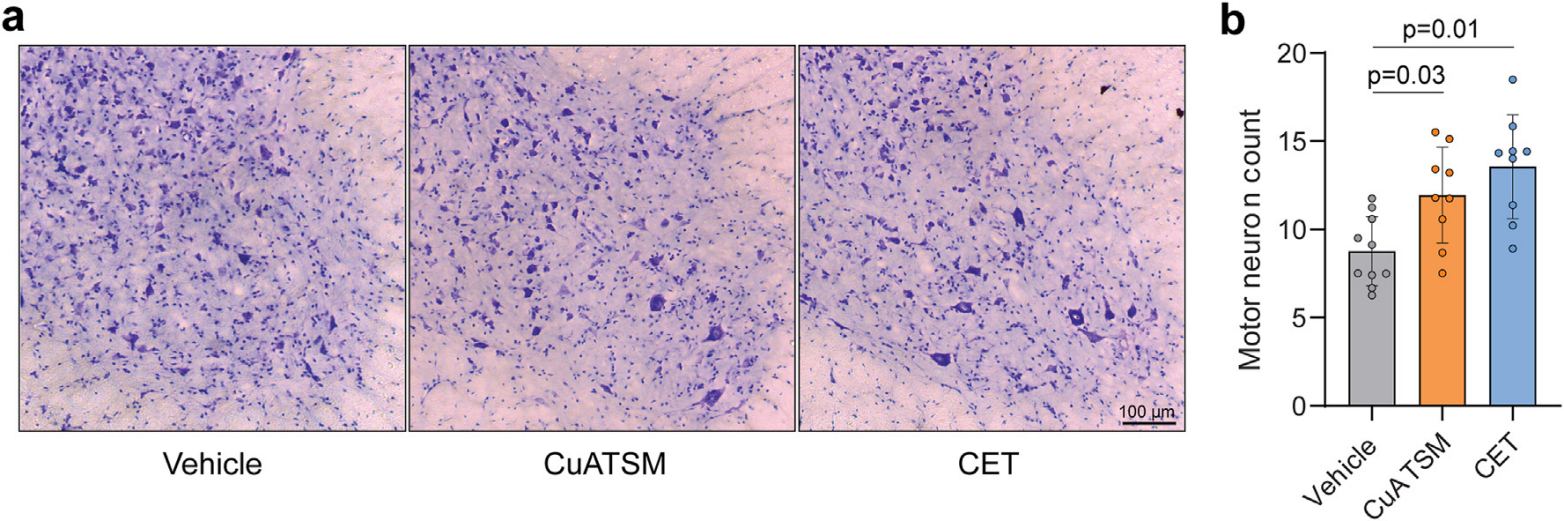
**CuATSM and CET treatment provides neuroprotection. (a)** Representative micrographs of thionin acetate-stained ventral horn lumbar spinal cord sections and **(b)** motor neuron counts from vehicle-, CuATSM- and CET-treated SOD1^G93A^ mice. Data shown are mean ± SD (*n* = 9–10/treatment). One-way ANOVA was used to compare relative differences between vehicle-, CuATSM- and CET-treated SOD1^G93A^ mice.

CuATSM has repeatedly shown to protect against motor neuron loss in SOD1 mouse models of ALS.^8^^,^^10^^,^^11^^,^^18^ In line with these findings, motor neuron counts within the lumbar ventral horn revealed CuATSM-treated SOD1^G93A^ mice had a 36% increase in motor neuron number compared to vehicle-treated mice (Fig. 6a–b). Similarly, CET-treated SOD1^G93A^ mice displayed a 55% increase in motor neuron number compared to vehicle-treated mice, whilst negligible differences were observed between CuATSM- and CET-treated mice.

## Discussion

Polytherapies are an increasingly favourable clinical approach to treat a wide variety of diseases. Here, we utilised a polytherapy approach to investigate if using a combination of CuATSM, ebselen and telbivudine, provides increased beneficial outcomes compared to the established therapeutic properties of CuATSM monotherapy in various SOD1 ALS models. Our results demonstrate that CET polytherapy reduces SOD1 inclusion formation, increases SOD1 maturation and provides neuroprotection, compared to CuATSM monotherapy in NSC-34 cells overexpressing human SOD1^G93A^. *In vivo* CET administration delayed disease onset, improved motor function and extended survival compared to both vehicle- and CuATSM-treated SOD1^G93A^ mice. Furthermore, CET increased soluble SOD1 levels and the proportion of disulfide bonded SOD1 and reduced misfolded SOD1 accumulation compared to vehicle-treated mice, which was not observed in CuATSM-treated mice. These results provide encouraging *in vitro* and *in vivo* evidence for a polytherapy approach in the treatment of *SOD1*-associated ALS.

The individual components of our CET polytherapy approach have previously demonstrated efficacy in *SOD1*-associated ALS models,^8^^,^^10^^,^^18^^,^^19^^,^^21^^,^^23^^,^^46^ supporting their use as single drug formulations. These studies highlight the potential each small molecule has to provide therapeutic benefit and reduce SOD1 toxicity through discreet mechanisms associated with mutant SOD1 misfolding. Utilising a 3D checkerboard approach, we demonstrate that CuATSM, ebselen and telbivudine in combination, reduces SOD1^G93A^ inclusion formation, increases the proportion of SOD1^G93A^ dimer formation, and cell survival compared to the highest concentration of CuATSM alone. This ability for the small molecules, particularly CuATSM and ebselen, to increase SOD1 maturation and reduce aggregation is in line with previous work supporting their on target engagement with SOD1.^8^^,^^10^^,^^18^^,^^21^^,^^22^^,^^46^^,^^47^ Furthermore, it should also be noted that the concentration of CuATSM used in our *in vitro* CET polytherapy studies was over two-fold lower than the highest concentration of CuATSM tested alone, and over three-fold lower than those that have previously shown *in vitro* efficacy.^19^^,^^20^ This is important as we have previously shown that relatively high concentrations of CuATSM can cause cell death *in vitro* and lead to adverse events in mice.^13^^,^^38^ This is an encouraging aspect of a broader polytherapy approach, in particular CuATSM polytherapy, as these *in vitro* results demonstrate that CuATSM can be used at lower concentrations in combination with ebselen and telbivudine, whilst producing similar or enhanced therapeutic effects.

Considering CET showed encouraging efficacy in cell culture, we moved to examine *in vivo* CET administration to ensure our approach was tolerable and reached the CNS. A major limitation of *in vivo* drug development for neurological diseases, is the ability of small molecules to cross the blood brain barrier at tolerable doses. Here, we show that when delivered in combination, CuATSM, ebselen and telbivudine are capable of reaching the CNS and are tolerable following sub-chronic oral administration. Whilst a toxicity profile of telbivudine has been extensively investigated across multiple species, with minimal effects at doses >10 times that used in the present study,^48^ to our knowledge no one has examined ebselen in this context. At the dosage used in the present study, sub-chronic CET administration in wild-type mice showed no adverse effects or elevated liver enzymes, indicating a relatively safe preclinical profile.

The SOD1^G93A^ mouse model is one of the most well characterised models of *SOD1*-associated ALS. We and others have demonstrated that CuATSM administration can modify disease progression and increase survival in the SOD1^G93A^ and other SOD1 mouse models.^8^, ^9^, ^10^, ^11^, ^12^, ^13^ In the present study, we provide further evidence that CuATSM delays symptom onset, improves motor function and extends survival compared to vehicle-treated SOD1^G93A^ mice. Furthermore, we show that CET polytherapy further delays symptom improves motor function and extends survival compared to CuATSM monotherapy. However, it should be noted that this was driven by the extended survival females CET-treat mice. Sex-specific disease progression and treatment effects in the SOD1^G93A^ mice have been repeatedly reported. Interestingly, an independent study, performed by the ALS Therapeutic Development Institute, reported CuATSM (30 mg/kg/day) extended survival in male SOD1^G93A^ mice.^9^ Whilst, we observed increased survival in both male and female mice at 50 mg/kg/day, the present results indicate the addition of ebselen and telbivudine have more profound effects on survival in female mice, compared to males, possibly as CuATSM has a larger therapeutic effect in male mice. However, this notion requires further investigation, particular of the sex-specific effects of each individual small molecule. Ebselen treatment has previously been shown to delay the age to reach maximum body weight (an indicator of disease onset) compared to untreated SOD1^G93A^ mice. Although, the aforementioned study reported negligible changes in motor impairment or survival of ebselen-treated mice, potentially due to using an estimated >300 times lower dose compared to that used in the present study.^21^ In addition, telbivudine has been shown to rescue SOD1 toxicity in a zebrafish model.^23^ The focus of this work was to assess if CuATSM therapy could be improved when used as a polytherapy with ebselen and telbivudine. Whilst, we did not assess the individual *in vivo* effects of ebselen and telbivudine alone in the SOD1^G93A^ mouse model, it will be important to assess if individual administration of these compounds can provide any therapeutic advantage. In particular, ebselen and ebselen-derivatives have shown to delay disease onset in SOD1^G93A^ mice,^21^^,^^46^ however they have not shown to extend survival. In addition, understanding the mechanism(s) of action, not only of the individual compounds, but potential synergies may open further therapeutic strategies.

The present study matched mice for sex, age and litters across treatment groups, as these have previously shown to be confounding variables in the SOD1^G93A^ mouse model.^34^ However, the present post-mortem analysis was performed on mice obtained from the efficacy study. Despite CET polytherapy treated mice being significantly aged, particularly compared to vehicle-treated mice, we still observed that CET increased soluble SOD1 levels, increased the proportion of disulifide-bonded SOD1 and reduced misfolded SOD1 accumulation. The initial notion behind this formulation was based on the compounds abilities to promote SOD1 folding and prevent aggregation however, it will be important to determine if increasing the levels of mature SOD1 or reducing the misfolded load underlies the therapeutic benefits. Furthermore, over 230 ALS-linked SOD1 variants have been identified, with all of them differentially impacting components of SOD1 maturation. It is important to note that CET polytherapy is likely to have minimal effects for patients carrying truncation variants as often large sections of the protein where CuATSM, ebselen or telbivudine act, are altered or removed. However, CuATSM, ebselen and telbivudine have been independently shown to suppress oxidative stress,^15^^,^^49^^,^^50^ gliosis,^10^^,^^15^^,^^51^ and ferroptosis,^16^^,^^52^ pathways associated with both sporadic- and familial-ALS,^53^ which may also underlie the therapeutic efficacy observed in CET-treated mice.

Collectively, the present study provides evidence that CET polytherapy confers improved benefits compared to treatment with CuATSM alone in models of *SOD1*-associated ALS. Whilst, the CET improvement in SOD1^G93A^ mouse survival was confined to females and relatively modest (10% increase compared to vehicle), in particular compared to Tofersen (22% increase compared to vehicle-treated SOD1^G93A^ mice^54^), the present data supports a notion of employing a polytherapy as a potential treatment approach for ALS and other proteinopathies. There has been a significant increase in the number of high-throughput drug screens in an effort to identify potential treatments for ALS.^55^ Whilst a large majority of studies progress a limited subset of candidate compounds down the pipeline, it may be of potential benefit to test a matrix of candidates to find those with additive or synergistic interactions on the desired phenotype. This would be particularly important whereby compounds have a relatively high degree of toxicity or may work in an independent manner. Furthermore, future polytherapy investigations should not be restricted to a single target (i.e., SOD1) and potentially target multiple aspects of ALS pathology such a gliosis, neurotrophic support, neuronal excitability, endoplasmic reticulum stress and apoptosis.

Recently, the *SOD1* antisense oligonucleotide (ASO), Tofersen, was approved for the treatment of *SOD1* ALS. Whilst Tofersen reduced plasma neurofilament light chain levels, there was negligible improvements in clinical end points after 24-weeks of treatment, whilst a three year longer term open label extensions have shown more promising results.^56^ However, Tofersen administration in patients with *SOD1* ALS was shown to only reduce SOD1 levels in cerebrospinal fluid by approximately one-third, indicating a large pool of mutant *SOD1* escapes ASO-mediated degradation, is continually translated and likely causes continual toxicity. It would be of potential interest to investigate if *SOD1* ASO delivery, in combination with CET therapy, may reduce this pool of potentially misfolded SOD1 further, particularly in the early stages of ASO treatment to and improve therapeutic outcomes.

Together, this data provides both *in vitro* and *in vivo* proof-of-concept evidence that the polytherapy use of these small molecules can aid SOD1 folding and reduce accumulation of toxic species in, providing therapeutic benefits in SOD1 models. CuATSM treatment has shown to slow disease progression when administered after disease onset in SOD1 mouse models.^10^^,^^12^ In an effort to develop this treatment paradigm further, it will be important to investigate administration following disease onset to recapitulate the typical clinical treatment scenario. However, the data presented here builds on previous data indicating a polytherapy approach targeting SOD1 maturation can provide therapeutic advantages compared to monotherapy with one of the most promising compounds to date, CuATSM. These findings support a role for polytherapy as a potential strategy for *SOD1* ALS, and deliver a foundation to further identify more advantageous therapeutic options for ALS.

## Contributors

Conceptualisation: J.S.L and J.J.Y. Investigation: J.S.L, M.B.L, N.E.F, R.B, A.D, F.D, C.G.C, J.G, L.E.M, H.E, P.J.C, P.S.D, L.M and J.J.Y. Data Curation: J.S.L, M.B.L, N.E.F, R.B, A.D, F.D, L.M and J.J.Y. Formal Analysis: Writing-original draft: J.S.L, M.B.L. and N.E.F Writing-review and editing: J.S.L, M.B.L, N.E.F, R.B, A.D, F.D, C.G.C, J.G, L.E.M, H.E, P.J.C, P.S.D, L.M and J.J.Y. Visualisation: J.S.L, M.B.L, N.E.F and R.B. Project administration: J.S.L, M.L.B and J.J.Y. Supervision: J.S.L, H.E, L.M and J.J.Y. Funding acquisition: P.J.C, P.S.D and J.J.Y. J.S.L, M.B and N.E.F have access to and verified the underlying data. All authors read and approved the final version of the manuscript.

## Data sharing statement

All data is available in the main text or Supplementary materials. Raw data, material or methods used or produced in this study can be shared for research purposes upon request to the corresponding author.

## Declaration of interests

P.S.D is named as inventor on intellectual property that relates to this research has been licenced from the University of Melbourne to Collaborative Medicinal Development. Collaborative Medicinal Development licenced intellectual property pertaining to CuATSM from the University of Melbourne where PJC is an employee but not a beneficiary of the licence agreement. P.J.C is an unpaid consultant for Collaborative Medicinal Development LLC. J.S.L received grant funding from Molecular Horizons and the University of Wollongong in the form of a Collaboration Grant (M2024). M.L.B, N.E.F, R.B, A.D, F.D, C.G.C, J.G, L.E.M, L.M and J.J.Y declare no conflicts of interest.

## Appendix ASupplementary data

Supplementary Figs. S1–S3

Supplemental western blots
