# Supplementary material for: A polytherapy approach demonstrates therapeutic efficacy for the treatment of SOD1 associated amyotrophic lateral sclerosis
Source: eBioMedicine. 2025 Apr 12;115:105692. doi: 10.1016/j.ebiom.2025.105692 (PMC12018197; doi:10.1016/j.ebiom.2025.105692)
Supplement: Supplementary Figs. S1–S3 [file mmc1.docx]

**A polytherapy approach demonstrates therapeutic efficacy for the treatment of**

***SOD1* familial amyotrophic lateral sclerosis**

Jeremy S. Lum^1,2*^, Mikayla L. Brown^1^, Natalie E. Farrawell^1^, Rachael Bartlett^1^, Christen G. Chisholm^1^, Jody Gorman^1^, Anthony Dosseto^3^, Florian Dux^3^, Lachlan E McInnes^4^, Heath Ecroyd^1^, Luke McAlary^1^, Peter J Crouch^5^, Paul S. Donnelly^4^ and Justin J. Yerbury^1^

**
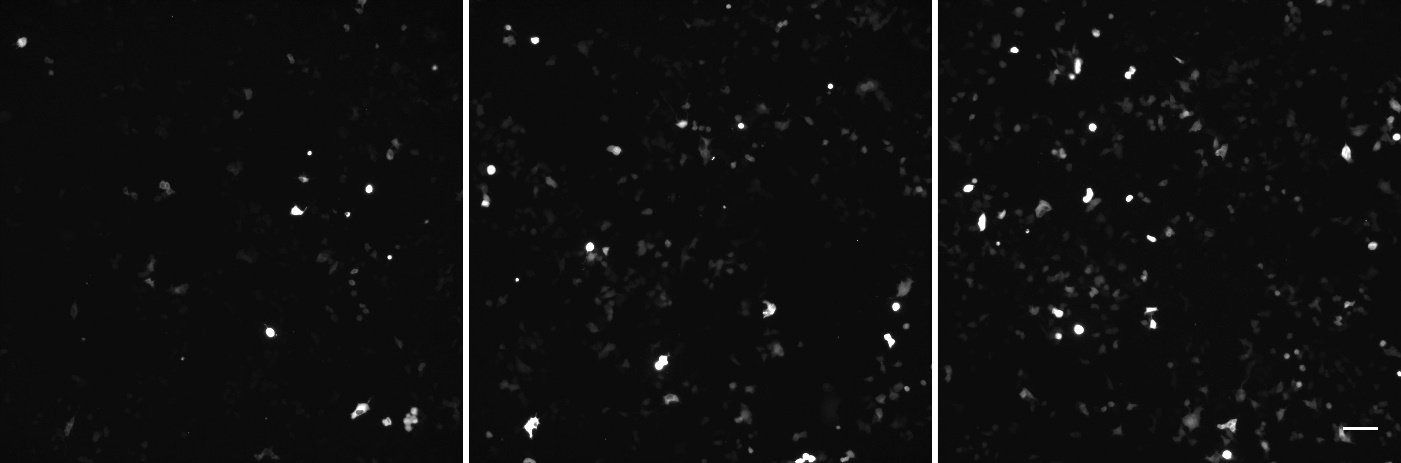
**

**Supplementary figure 1:** NSC-34 cells were transiently transfected with SOD1^G93A^-EGFP constructs, treated with varying concentrations of CuATSM, ebselen and telbivudine and imaged 48 h following treatment. Representative images of NSC-34 cells transfected with SOD1^G93A^-EGFP and treated with either vehicle (0.5% DMSO (v/v); left), 0.5 µM CuATSM (middle) and CET (0.25 µM CuATSM, 20 µM ebselen and 62.5 µM telbivudine; right) for 48 h. Scale bar represents 100 μm.

**
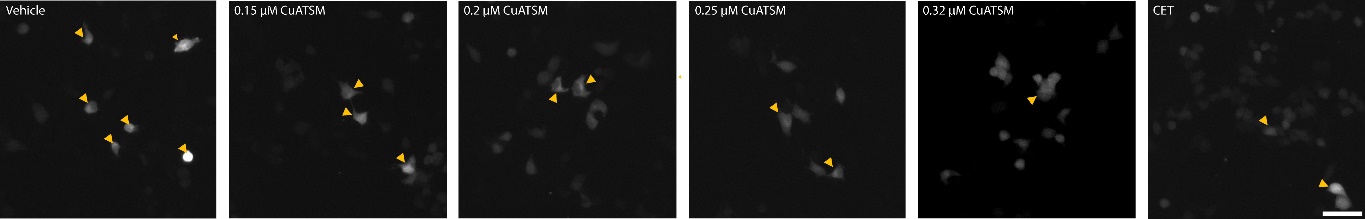
**

**Supplementary figure 2:** Representative images of NSC-34 cells transfected with SOD1^G93A^-EGFP and treated with either vehicle (0.5% DMSO (v/v)), various concentrations of CuATSM or CET (0.25 µM CuATSM, 20 µM ebselen and 62.5 µM telbivudine; right) for 48 h. Scale bar represents 50 μm. Arrows indicate cells with inclusions.


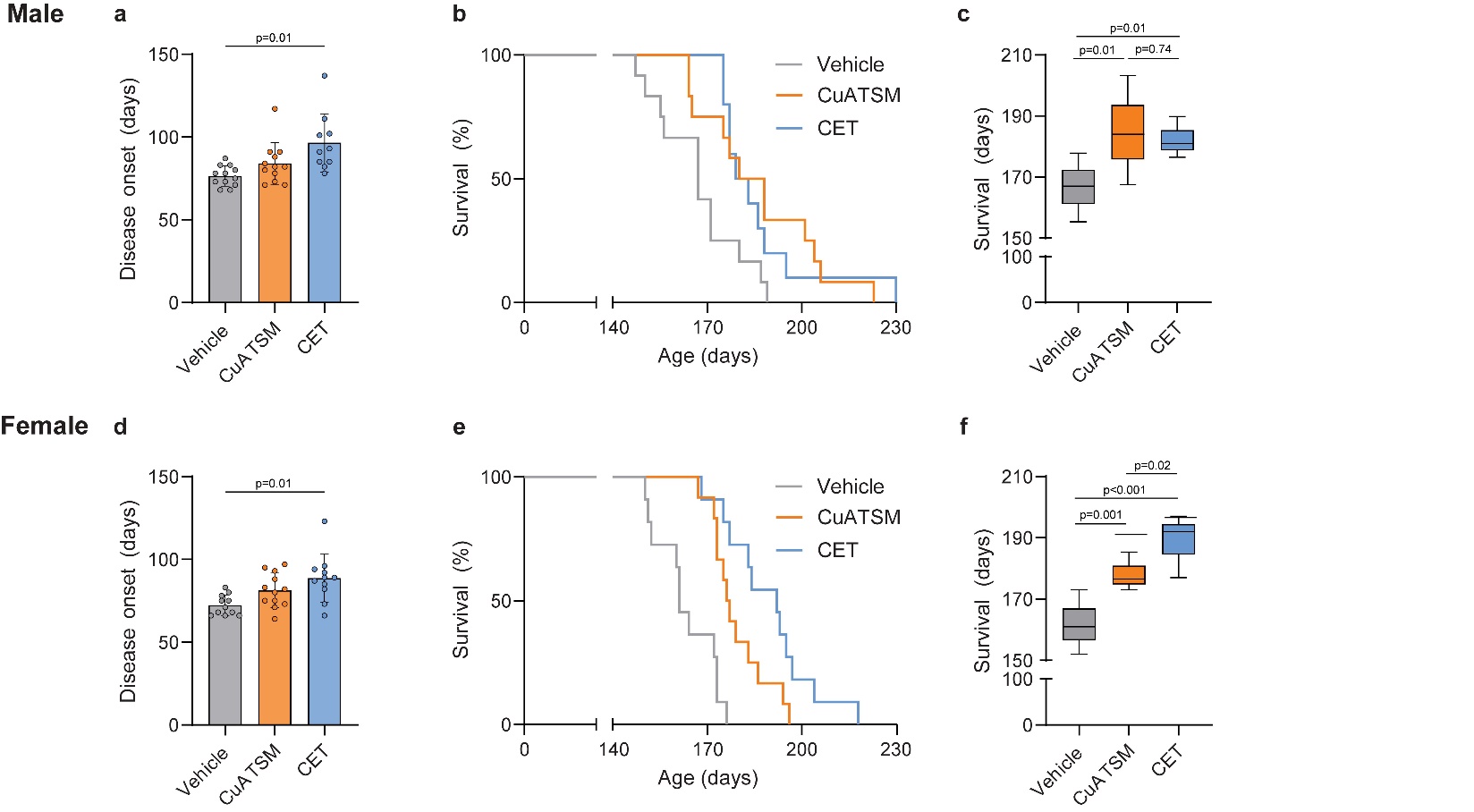
**Supplementary figure 3:** The sex-specific effect of daily oral gavage administration of either vehicle, CuATSM or CET on (**a)** male and (**d**) female age of disease onset (defined as attaining a neurological score of 1), (**b**) male and **(e**) female Kaplain-Meier plot and (**c**) male and (**f**) female survival. (**a** and **d**) Data is shown as mean ± SD (*n*=10-12 per treatment group). (**c** and **f**) Data is shown as median ± IQR.
