# Supplementary figures and images for: A polytherapy approach demonstrates therapeutic efficacy for the treatment of SOD1 associated amyotrophic lateral sclerosis
Source: eBioMedicine. 2025 Apr 12;115:105692. doi: 10.1016/j.ebiom.2025.105692 (PMC12018197; doi:10.1016/j.ebiom.2025.105692)

**Supplemental western blots and gels**


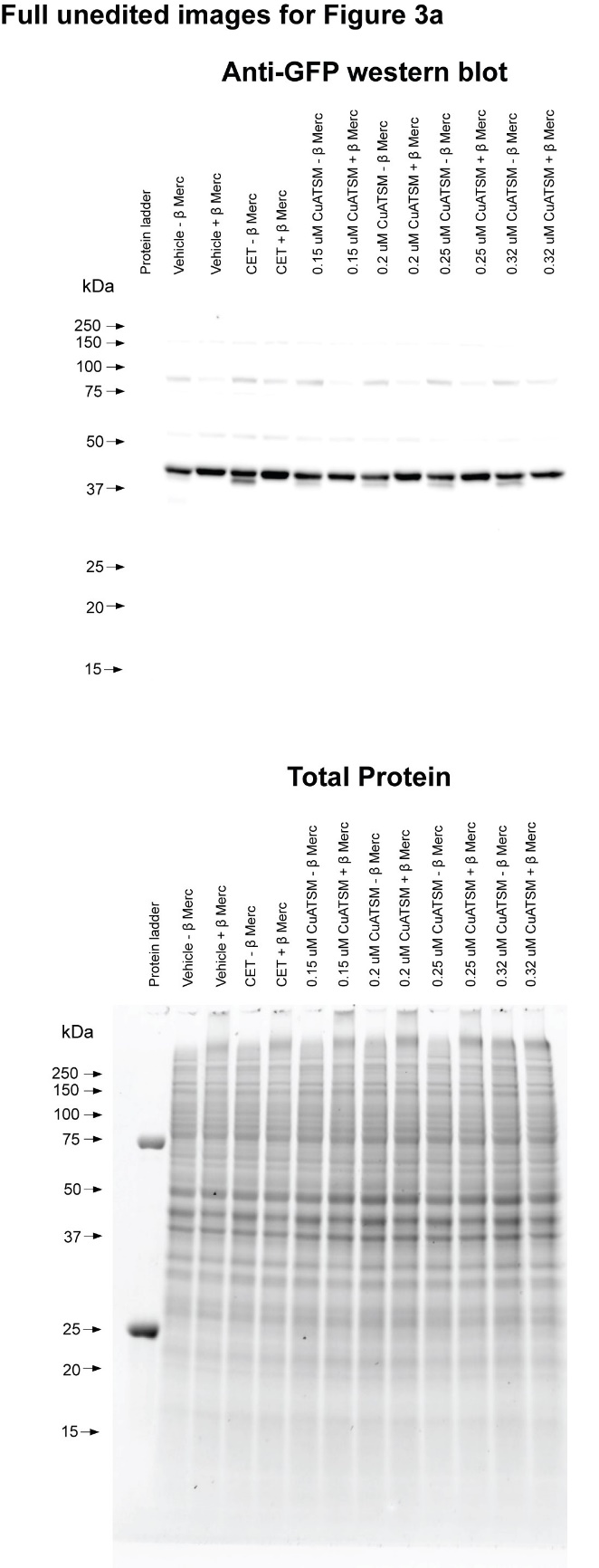


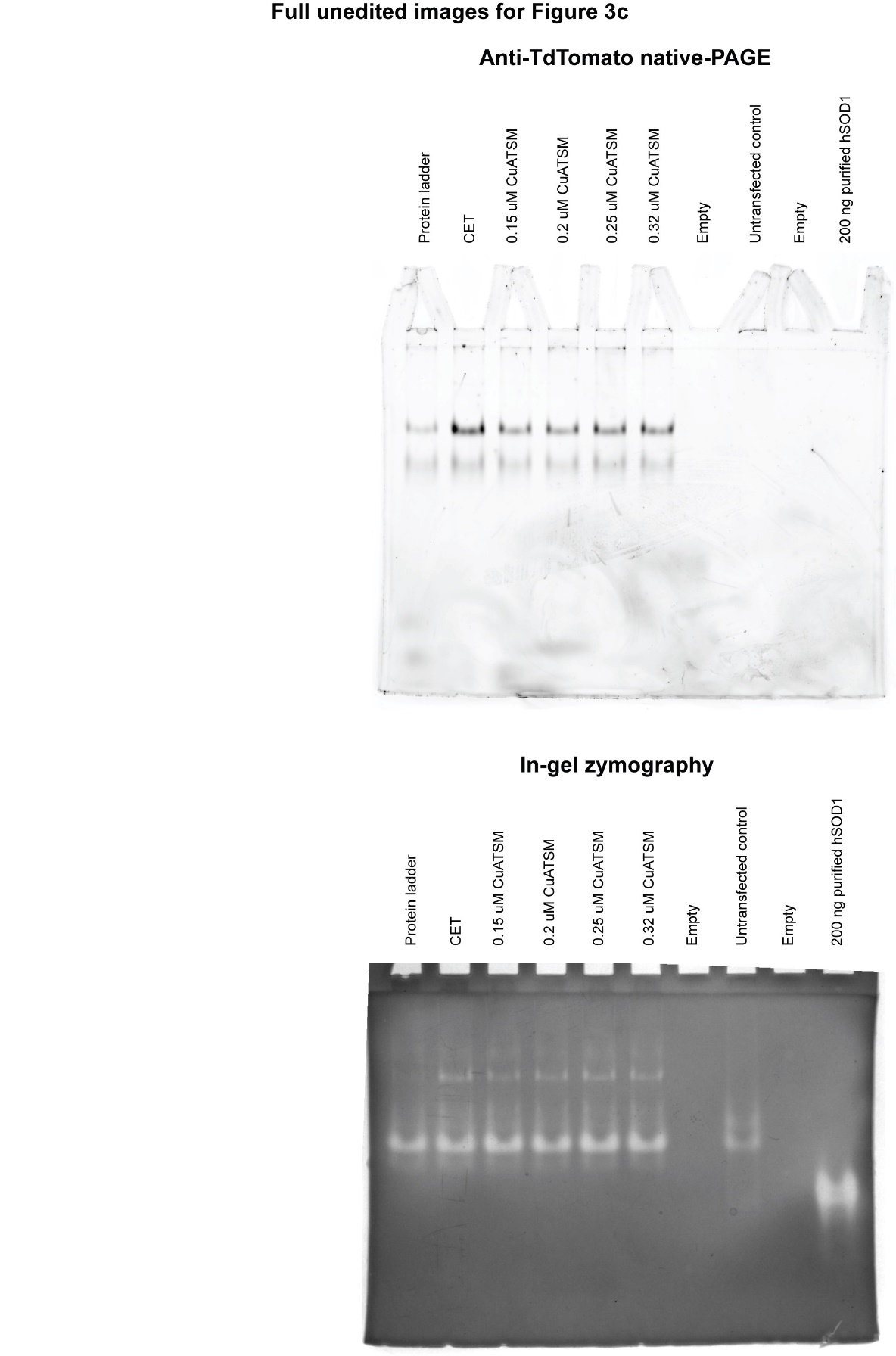


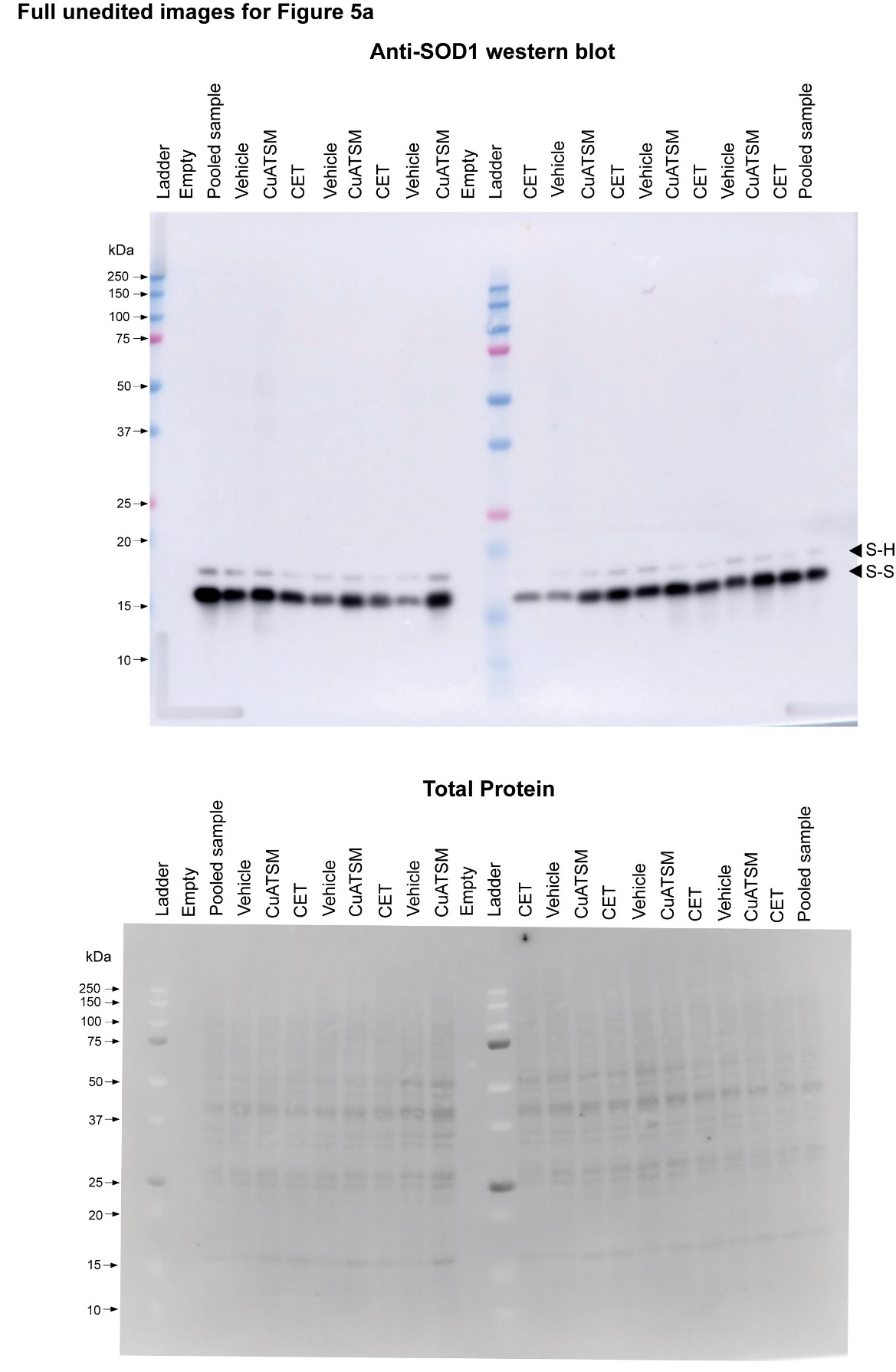


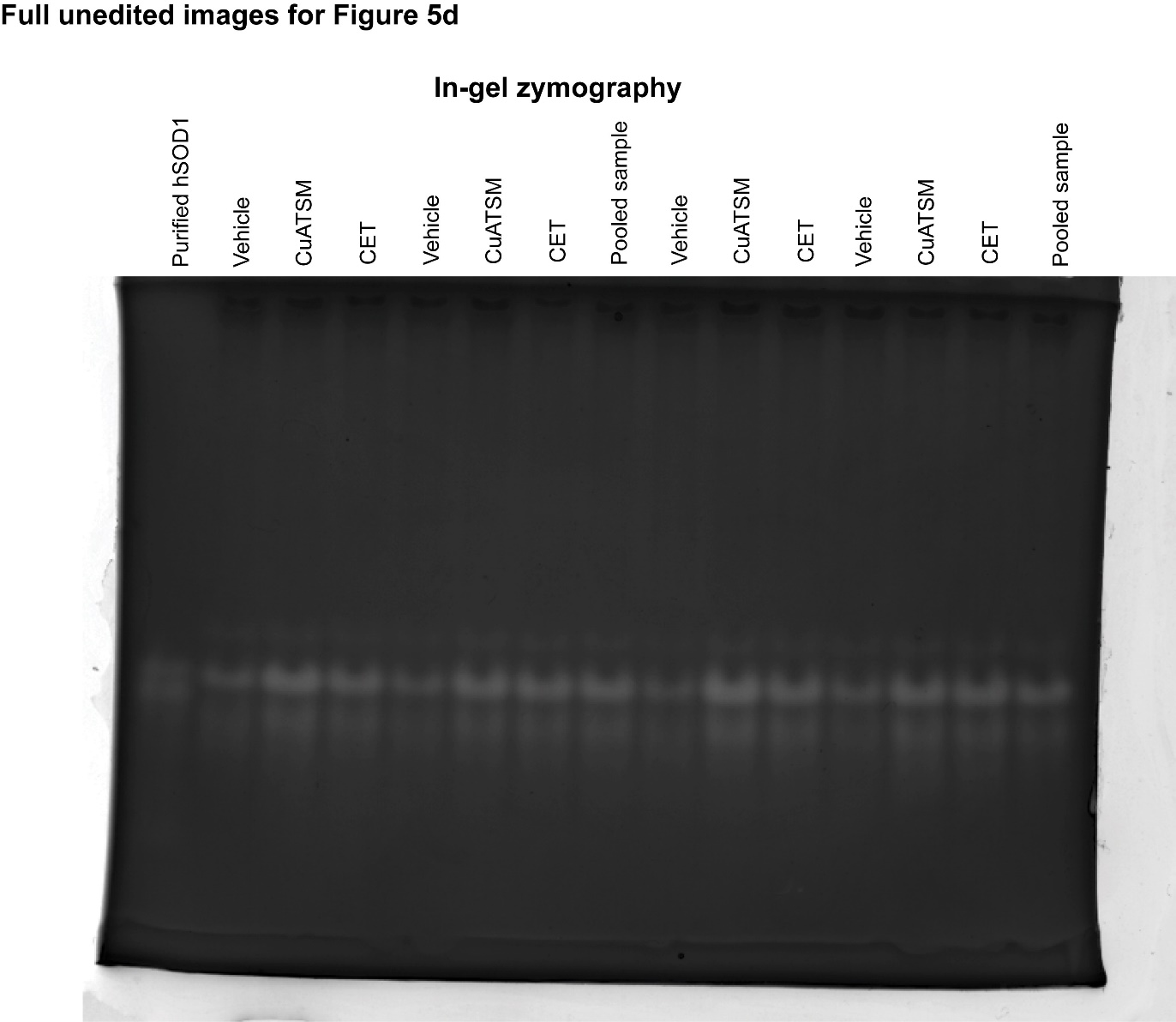


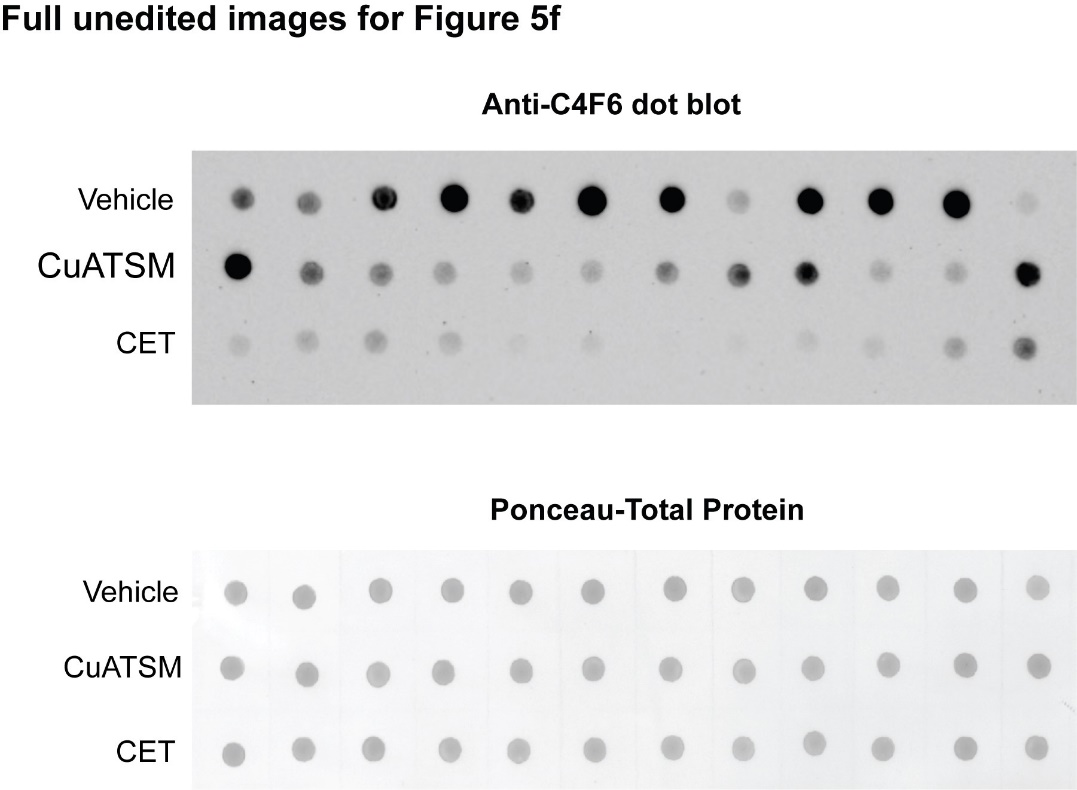

Supplement: Supplemental western blots [file mmc2.docx]
